# Augmenting hippocampal–prefrontal neuronal synchrony during sleep enhances memory consolidation in humans

**DOI:** 10.1038/s41593-023-01324-5

**Published:** 2023-06-01

**Authors:** Maya Geva-Sagiv, Emily A. Mankin, Dawn Eliashiv, Shdema Epstein, Natalie Cherry, Guldamla Kalender, Natalia Tchemodanov, Yuval Nir, Itzhak Fried

**Affiliations:** 1 Department of Neurosurgery, University of California, Los Angeles, Los Angeles, CA USA; 2 Sagol School of Neuroscience, Tel Aviv University, Tel Aviv, Israel; 3 Department of Neurology, University of California, Los Angeles, Los Angeles, CA USA; 4 Department of Physiology and Pharmacology, Sackler School of Medicine, Tel Aviv University, Tel Aviv, Israel; 5 Department of Biomedical Engineering, Faculty of Engineering, Tel Aviv University, Tel Aviv, Israel; 6 Present Address: Center of Neuroscience, University of California, Davis, Davis, CA USA

**Keywords:** Consolidation, Non-REM sleep, Neural circuits

## Abstract

Memory consolidation during sleep is thought to depend on the coordinated interplay between cortical slow waves, thalamocortical sleep spindles and hippocampal ripples, but direct evidence is lacking. Here, we implemented real-time closed-loop deep brain stimulation in human prefrontal cortex during sleep and tested its effects on sleep electrophysiology and on overnight consolidation of declarative memory. Synchronizing the stimulation to the active phases of endogenous slow waves in the medial temporal lobe (MTL) enhanced sleep spindles, boosted locking of brain-wide neural spiking activity to MTL slow waves, and improved coupling between MTL ripples and thalamocortical oscillations. Furthermore, synchronized stimulation enhanced the accuracy of recognition memory. By contrast, identical stimulation without this precise time-locking was not associated with, and sometimes even degraded, these electrophysiological and behavioral effects. Notably, individual changes in memory accuracy were highly correlated with electrophysiological effects. Our results indicate that hippocampo–thalamocortical synchronization during sleep causally supports human memory consolidation.

## Abstract (web summary)

Geva-Sagiv et al. performed real-time closed-loop intracranial stimulation in human sleep. Brief prefrontal pulses, precisely timed with MTL slow-wave active periods, led to correlated enhancements in sleep oscillation coupling and memory accuracy.

## Main

For decades, it has been demonstrated that sleep plays an important role in long-term memory consolidation^1–4^. Systems-level memory consolidation theory posits that the initial phase of the formation of a declarative memory trace (that is, memories that are accessible to conscious recollection, such as memory for facts and events^5^) is primarily supported by the hippocampus. Over time, declarative memory representations become increasingly dependent upon the neocortex (a ‘two-stage’ model)^4,6,7^. A central notion in this model is that embedding novel information in the neocortex relies on offline reactivation of acquired information by the hippocampus around ripple events, primarily during slow-wave sleep^4,8^. Ripples (brief oscillatory events with frequencies of ~80–120 Hz in humans) occur in and around the hippocampus in the MTL, as well as in the neocortex^9,10^. Extensive research in rodents has established the role of hippocampal ripples in memory consolidation^11^ and their widespread impact on neocortical activities during sleep^12,13^. Slow waves (<4 Hz) reflect regionally synchronous alternations between active states of membrane depolarization and spiking, and inactive states of hyperpolarization and neuronal quiescence^14,15^. According to the active system consolidation framework, slow-wave active states serve as a temporal frame for offline consolidation via synchronization of thalamocortical sleep spindles (9–16 Hz) and ripple oscillations^4,16,17^. Thus, sleep-dependent memory consolidation is believed to be mediated by coordinated oscillations across hippocampus, thalamus and neocortex.

Most evidence supporting this theory stems from correlative studies, performed either noninvasively in humans^1,4,18,19^ or with neuronal recordings in rodents^20–24^. Causal manipulations of ripple activities in rodents established that ripples are necessary for optimal memory consolidation^25–27^. To date, only a few studies have provided causal support for the role of coordinated hippocampal–neocortical interactions during sleep in mediating memory consolidation^20,21^. In humans, there is evidence highlighting extensive hippocampal–neocortical interactions during sleep^13,28,29^, but direct causal evidence linking these interactions with memory consolidation is currently missing^30^. Here, we designed a closed-loop stimulation protocol to dynamically enhance the temporal coupling between MTL ripples, neocortical slow waves and thalamocortical spindles during non-rapid eye movement (NREM) sleep to directly test the role of their temporal coupling in overnight consolidation of declarative memory.

## Results

**Fig. 1:**
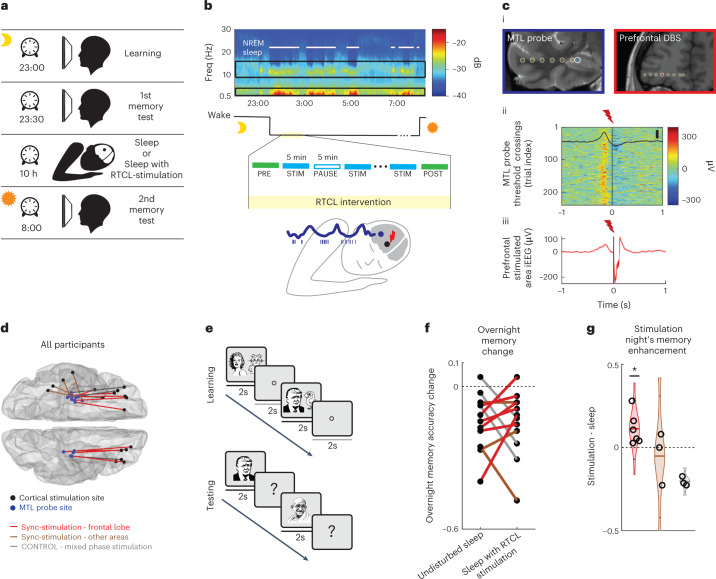
Neocortical stimulation synchronized to medial temporal lobe sleep activity improves overnight recognition memory accuracy. **a**, Experimental design. Each individual participated in two overnight sessions (order counterbalanced), an undisturbed sleep session and another session with RTCL neocortical stimulation. Memory was assessed immediately following evening learning and following sleep. **b**, Top, representative spectrogram of iEEG during overnight sleep session (short-time Fourier transform; Methods). Black rectangles mark slow-wave (0.5–4 Hz) and sleep spindle (9–16 Hz) frequency bands used for NREM detection (white dots). Middle, RTCL intervention lasted 45–90 min with alternating 5-min stimulation (STIM) and PAUSE intervals. PRE interval (interval before the first stimulation block) is used for some analyses. Bottom, schematic of RTCL approach where MTL slow-wave active states (blue iEEG troughs, co-occurring with neuronal activity, bars) are used to trigger neocortical stimulation pulses (red). **c**, Representative RTCL input and DBS sites: (i) Coronal magnetic resonance (MR) images denoting iEEG electrode locations. Blue, RTCL input’s MTL location; red, prefrontal white-matter DBS location; yellow, iEEG contacts on same electrodes. (ii) Single-trial MTL probe iEEG signals (each row denotes a trial): increased voltage (warm colors) triggered stimulation in a neocortical site at *t* = 0 s (participant 2, *n* = 244 stimulations). Average and s.e.m. of the MTL probe signal are superimposed (black; scale bar, 100 μV). (iii) Average iEEG signal adjacent to the neocortical stimulation site, aligned to stimulation pulses. **d**, All pairs of MTL probe (blue) and stimulation site electrodes (black), overlaid on a standard (Montreal Neurological Institute (MNI)) brain template (*n* = 18 participants). Line color depicts stimulation type. Red, synchronizing stimulation (sync-stim) in prefrontal cortex; brown, sync-stimulation in temporal neocortical regions; gray, mixed-phase stimulation in prefrontal neocortex. **e**, Learning and memory paradigm presented image pairs of celebrities and animals, followed by recognition memory testing (Methods). **f**, Overnight change in recognition memory accuracy following undisturbed sleep versus sleep with RTCL stimulation. Line colors as in **d**. **g**, Within-participant difference in overnight recognition memory accuracy between intervention night and undisturbed sleep. All participants with sync-stimulation in orbitofrontal cortex (red) show superior performance in stimulation nights (*P* = 0.01, binomial test), while none of mixed-phase stimulation participants (gray) do. Source data

**Extended Data Fig. 1:**
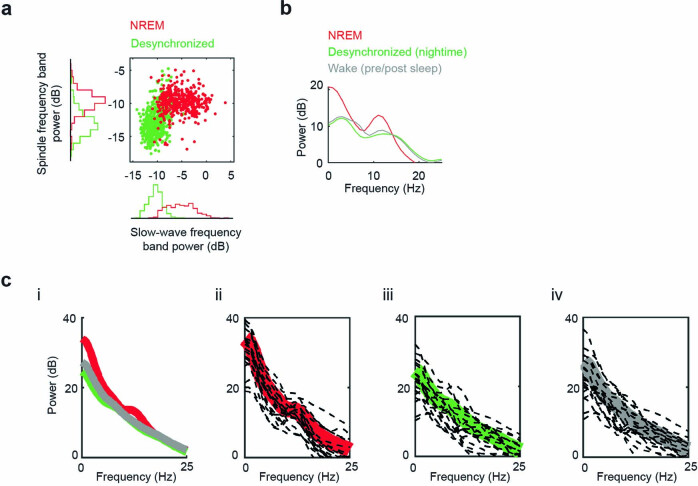
Automated scoring of NREM sleep intervals based on iEEG. An example of overnight NREM detection performed on orbitofrontal cortex iEEG activity, used for sleep scoring in participant #3 (full spectrogram: Fig. 1b). (**a**) Scatter plot of spindle power (9–16 Hz) versus slow-wave power (0.5–4 Hz). Each dot marks a 30 sec epoch, and its color denotes scoring as NREM (red) or desynchronized (REM sleep/wakefulness, green), according to the maximum posterior probability of a 2-component Gaussian mixture fit to the entire dataset. (**b**) iEEG power spectrum for each vigilance state for participant #3. Red: NREM sleep. Green: overnight desynchronized. Gray: unequivocal wakefulness periods occurring before or after the overnight sleep session. Note that iEEG power spectrum during overnight desynchronized states (green) resembles that found for unequivocal wakefulness (gray). (**c**) (i) Grand mean iEEG power spectra over all participants for all sleep/wake stages (n = 19 overnight sessions). Colors as in b. (ii-iv) Solid lines show the mean spectra across all patients; Dashed lines denote power spectrum per patient for NREM sleep (ii), overnight desynchronized states(iii), and unequivocal wakefulness periods(iv). Source data

**Extended Data Fig. 2:**
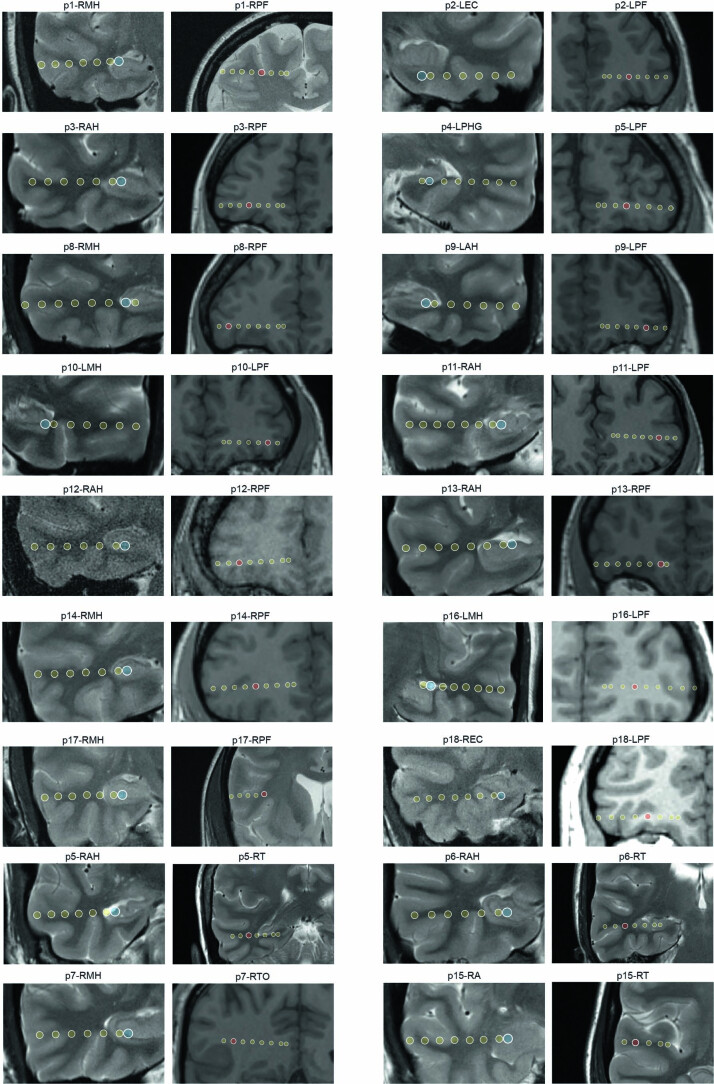
Location of MTL synchronization-probe and neocortical stimulation iEEG electrodes. For each participant (p1–18), two coronal MR images show the locations of the MTL synchronization-probe for closed-loop control (left image, blue circles) and neocortical stimulation site (right image, red circles, for bipolar stimulations the adjacent contact was used as reference). Yellow circles depict other iEEG contacts on the depth electrode. Title for each MR image: p = participant, number corresponds to participant-id in Supplementary Tables 1–4. Then the location of the highlighted iEEG contact. R = right, L = Left; PF = prefrontal cortex, T = temporal cortex, TO = Temporal-occipital cortex, AH = Anterior Hippocampus, MH = Middle hippocampus, EC = Entorhinal cortex, PHG = Parahippocampal gyrus. Note that participants 5–7,15 are shown at the bottom, as stimulation site was outside the prefrontal lobe.

**Extended Data Fig. 3:**
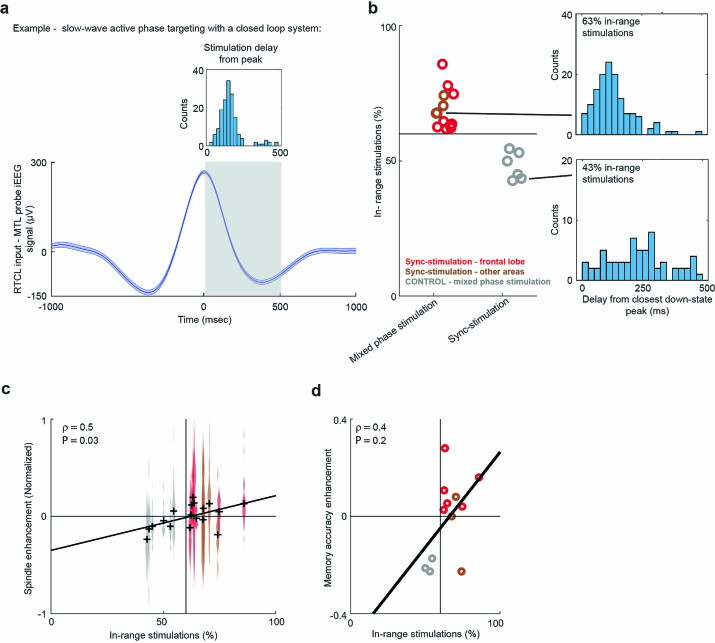
Closed loop system. **(a)** Example from participant #7 showing the average and SEM of MTL probe’s iEEG signal (blue trace, filtered between [0.5–4]Hz to highlight slow-wave activity), time-locked to the positive iEEG peak immediately preceding stimulation time (t = 0). Note that iEEG peak corresponds to the neuronal inactive slow-wave phase. Top inset: distribution of stimulation delays (n = 423 stimulation events during period highlighted by gray background) from iEEG slow-wave positive peak for this participant**. (b)** Our phase targeting method was based on detecting peaks in the iEEG signal and delivering stimulation at a pre-determined delay following the peak. We quantified the degree to which stimulations were delivered in phase with MTL active periods *post hoc*. All sync-stimulation patients (red, brown) had >60% stimulations delivered in the planned delay range, while mixed-phased patients (gray) had <45% stimulations within that range. Two subpanels on the right depict two representative distributions of stimulation delays in two patients – patient #18 from sync-stim group (top) and patient #1 from the mixed-phase group (bottom). **(c)** Individual immediate effect of spindle increase reveal significant positive correlation to the percentage of in-range stimulations (Spearman correlation: ρ = 0.51, *P = 0.027, n = 18 nights). The distribution across all iEEG contacts for each stimulation night is shown; black crosses mark the mean spindle enhancement in each subject. Red, sync-stimulation in prefrontal cortex. Brown, sync-stimulation in other neocortical regions. Gray, mixed-phase stimulation in prefrontal cortex. **(d)** Memory enhancement (as in Fig. 1g) is positively correlated with the percentage of in-range stimulations (Methods, Spearman correlation: ρ = 0.40, P = 0.19, n = 12 patients). Source data

Neurosurgical patients with pharmacoresistant epilepsy who were implanted with intracranial depth electrodes for clinical reasons (*n* = 18, ages 19–47 years, all fluent English speakers; Supplementary Table 1) provided written informed consent before participation in a study approved by the University of California, Los Angeles (UCLA) Institutional Review Board. The unique intracranial clinical setup allowed simultaneous recordings of intracranial electroencephalography (iEEG) and single-neuron activity in the MTL and distant neocortical sites. Electrophysiology was complemented by cognitive assessment, whereby participants were tested during two experimental nights (order counterbalanced): an intervention night and an undisturbed night (Fig. 1a and Supplementary Table 2). This within-participant design helped control for individual variability in clinical and memory profiles^31^. On the intervention night, real-time closed-loop (RTCL) stimulation was performed intermittently in 5-min blocks for a total of ~90 min during early NREM sleep (Methods, Fig. 1b, Extended Data Fig. 1 and Supplementary Table 3). One iEEG electrode in the MTL served as a synchronization probe for determining the timing of closed-loop control, while a second neocortical iEEG electrode served as the stimulation site (typically, in orbitofrontal cortex white matter (15 of 19 stimulation nights); Fig. 1c,d and Extended Data Figs. 2 and 3). Slow-wave activity in the MTL probe was monitored and analyzed in real time to trigger brief (50 ms) high-frequency (100 Hz) electrical stimulation events in the neocortical stimulation site roughly once every 4 s (Methods). The closed-loop intervention had two modes of operation (Methods and Extended Data Fig. 3)—either (i) ‘synchronized (sync) stimulation’ (Fig. 1c) or (ii) ‘mixed-phase stimulation’—which were applied in two separate groups of participants. Sync-stimulation involved neocortical stimulation that was time-locked to the MTL slow-wave active phase, aimed at synchronizing MTL with thalamocortical activities. During these active phases, corresponding to the iEEG negative peak (Fig. 1c)^32^, ripples occur more frequently and their prevalence is believed to be key for hippocampal–cortical communication^20,28,33,34^. During mixed-phase stimulation, performed in a separate group of participants, identical neocortical stimulations were applied but their timing was without regard to the MTL slow-wave phase (Methods and Extended Data Fig. 3). Given our previous work on local sleep oscillations, where MTL slow waves can be phase-shifted or even entirely independent from neocortical slow waves^32,35,36^, we hypothesized that sync-stimulation would be key in increasing hippocampo–neocortical coupling and that stimulating white-matter electrodes during sleep would allow local low-amplitude stimulation to affect wide territories^36,37^.

### Synchronized stimulation improved memory accuracy

**Extended Data Fig. 4:**
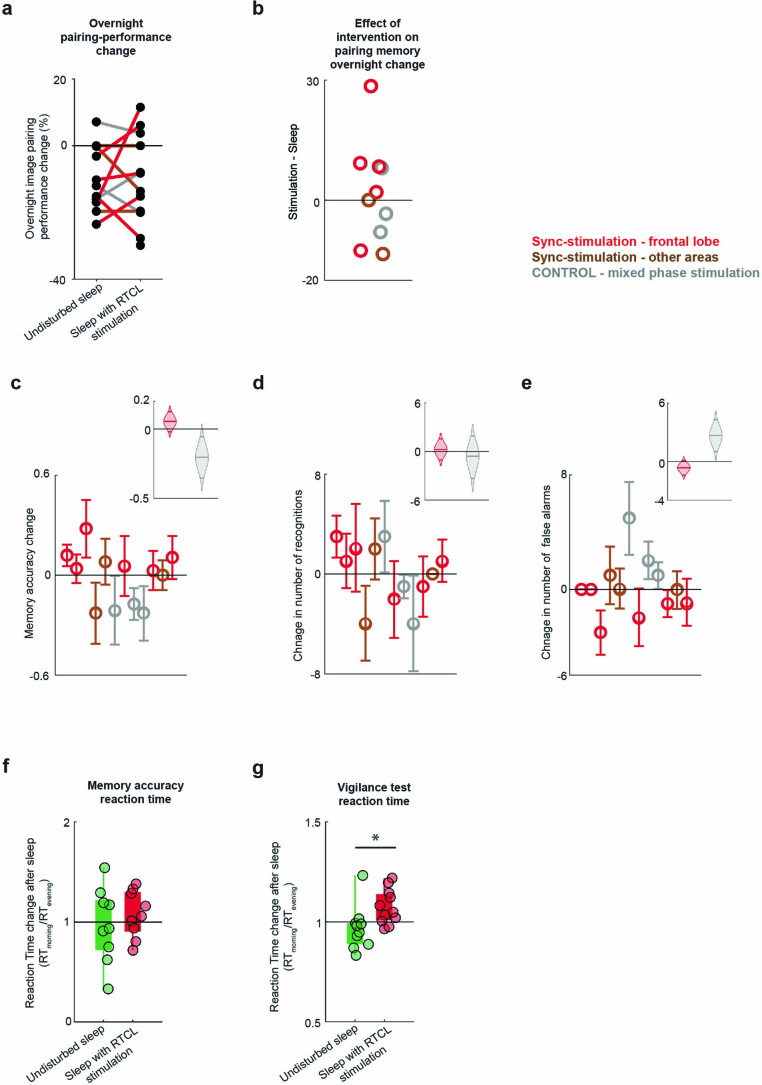
Behavioral measures. (**a**, **b**) **Association testing**: Participants were asked to recall the animal associated with every person they recognized from the learning session. Pairing index (PI; 100*number correct/number attempted). (**a**) Overnight change PI_Morning_-PI_Evening_ is plotted for participants who were tested following undisturbed sleep (left) and following a sleep with RTCL stimulation (right). Line color depicts stimulation type: red, synchronizing stimulation (n = 5, one participant chose not to complete the association test after undisturbed sleep night); brown, synchronizing stimulation, delivered in other regions (n = 2); gray, mixed-phase stimulation in prefrontal neocortex (n = 3). (**b**) Within-subject difference of overnight change in pairing success between intervention night and undisturbed sleep (difference between the dots in panel a): 5 of 7 participants with sync-stimulation (red, brown) showed either no change or superior performance in stimulation nights (Stimulation – Sleep > = 0), while only 1 of 3 participants with mixed-phase stimulation (gray) showed this effect. There were no significant changes in pairing success rates following RTCL stimulation relative to undisturbed nights. (**c**–**e**) Estimating effect size for recognition memory accuracy and its components (hit rate and false alarm rate): we performed bootstrapping (n = 1000) by selecting a random sample (with replacement) from each night’s image set and recalculating each memory evaluation measure on the subsampled data for each patient. The main panel displays the mean and standard deviation of the bootstrapped values for each patient, and the inset shows the distribution of the means across patients for each bootstrapping cycle, aggregating all sync-stim patients in red and mixed-phase patients in gray. (**c**) Recognition memory accuracy. The mean change in memory accuracy for the sync-stim averaged bootstrapped group trends toward improvement, but does not reach the 5% significance level, as the 95% confidence interval contains 0. (**d**) Correctly recognized images (hits). Each intervention had minimal effect on this measure. (**e**) Wrongly identified lures (false alarms). None of the frontal-lobe sync-stimulation subjects (red) exhibited an increase in the number of false alarms, while all mixed-phase subjects exhibited such an increase. The distribution of estimated means indicates a trend toward a decrease in false alarms for the sync-stimulation subjects and a significant increase in false alarm rate for the mixed-phase stimulation subjects. This suggests that the difference in performance following the two types of stimulation can be attributed to distinct effects on source memory. (**f**, **g**) Reaction time (RT) changes: (**f**) Change in mean RT for recognized images was not significantly different between intervention nights (red) and undisturbed nights (green) (n = 9, P = 0.65; Wilcoxon rank-sum test). (**g**) RT change on a separate psychomotor vigilance task (PVT, see Methods) shows significantly faster performance following undisturbed sleep than after sleep with RTCL stimulation (n = 11, P = 0.01; Wilcoxon rank-sum test). The bounds of the boxes (panels f, g) represent the interquartile range and whiskers extend between 1–99 percentiles. Source data

To assess the effects of the intervention on overnight memory consolidation, participants performed a visual paired-association task before sleep, learning 25 pairings between photos of famous people and animals (contextualized as ‘pet owners’ and their pets) on the evening before each experimental night (Fig. 1e and Methods). A different set of images was used each night. Two different measures assessed two separate aspects of memory performance. First, we evaluated recognition memory via responses to learned images and a set of lures, quantifying recognition memory accuracy as the difference between hit rate and false-detection rate (Methods). Second, we evaluated the successful pairing (association) of each person to their associated animal. Performance on these two measures was assessed four times for each participant (two time points × two nights), without any feedback on responses: in the evening (several minutes following learning) and the morning (following overnight sleep), separately around an intervention night and an undisturbed sleep night (Fig. 1a). Twelve participants completed this full cognitive testing suite, and six additional participants were only included in neurophysiological analyses (Methods and Supplementary Tables 2 and 3). In 6/6 participants receiving sync-stimulation in prefrontal cortex white matter (Fig. 1f,g), recognition memory accuracy following the intervention night was superior to that following undisturbed sleep (*P = 0.01 based on a binomial probability distribution; Methods and Extended Data Fig. 4c–e). Mixed results were observed for sync-stimulation delivered in other posterior neocortical regions (Fig. 1f,g; *n* = 3), and a trend for degraded performance was observed for participants who received mixed-phase stimulation (Fig. 1f,g; *n* = 3). Sync-stimulation did not reliably affect the pairing (association) accuracy (Extended Data Fig. 4a,b). We did not find significant correlation between baseline recognition memory accuracy on the first evening test and intervention efficacy (Spearman correlation; *n* = 9 sync-stimulation participants, ρ = 0.04, *P* = 0.9), suggesting memory improvements were not unique to participants with lower performance. Sync-stimulation did not significantly alter reaction times during memory recall compared with undistributed sleep (Wilcoxon rank-sum test: *P* = 0.65; Extended Data Fig. 4f). Conversely, overnight decrease in reaction times, representing an improvement in a visual psychomotor vigilance task (PVT; Methods^38^) was significantly lower following sleep with sync-stimulation as compared with undisturbed sleep (Extended Data Fig. 4g), indicating that memory improvement does not reflect an across-the-board improvement in behavioral performance. Therefore, memory accuracy improvement associated with sync-stimulation likely reflects an enhanced stabilizing effect of sleep to reduce forgetting^3,4^.

### Correlated enhancement of sleep spindles and memory accuracy

**Fig. 2:**
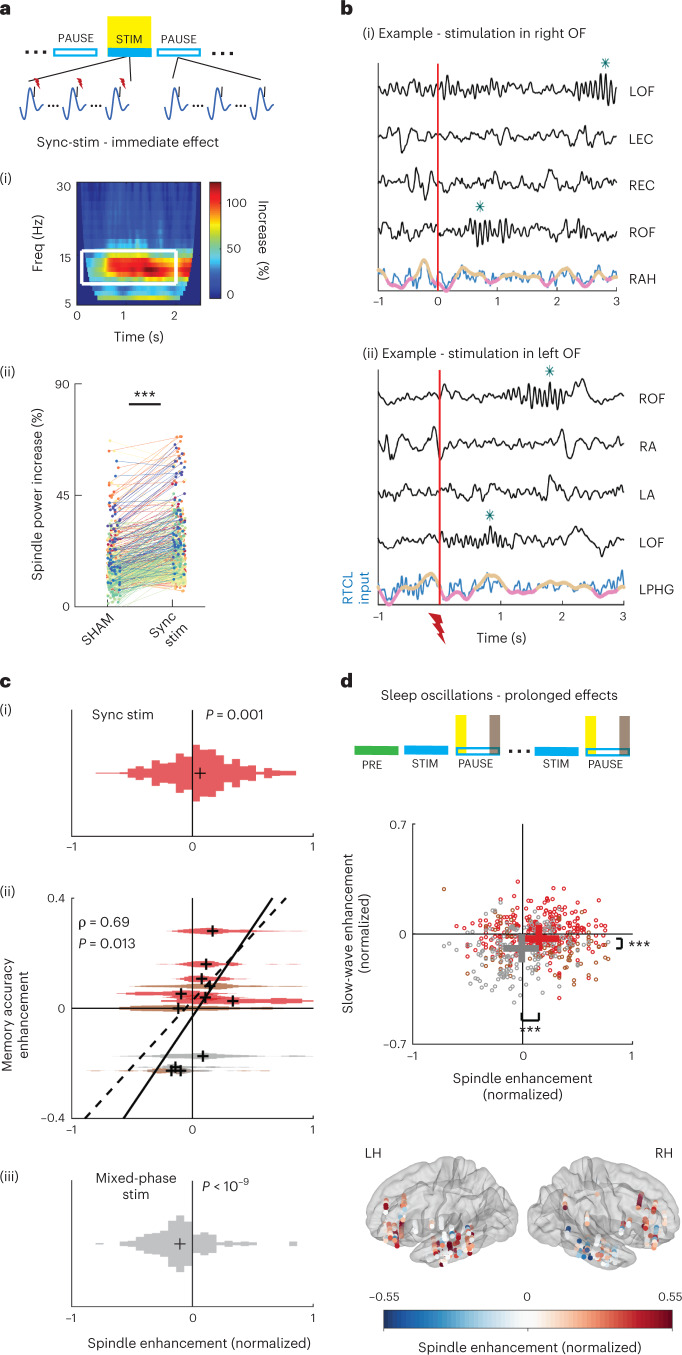
Synchronized stimulation enhancement of sleep spindles correlates with memory accuracy improvements. **a**, Immediate (<3 s) changes in sleep spindle activity during STIM blocks (yellow highlight) compared to sham intervals during PAUSE blocks. (i) Representative average time–frequency response (TFR; induced power) following stimulation in orbitofrontal cortex iEEG shows immediate increase in spindle power (9–16 Hz, white rectangle). (ii) Enhanced spindle power following sync-stimulation compared to sham stimulation (*n* = 565 iEEG electrodes). *P* = 1.4 × 10^−39^ via Wilcoxon paired signed-rank test. **b**, Representative spindles (blue asterisks) in simultaneously recorded iEEGs (black time-courses, *z*-scored for visualization) of two participants. Bottom time course (blue), MTL signal used for stimulation timing, superimposed with slow-wave-filtered (<2.5 Hz) signal showing active (pink) versus inactive (brown) phases; R, right; L, left; OF, orbitofrontal cortex; EC, entorhinal cortex; AH, anterior hippocampus; A, amygdala; PHG, parahippocampal gyrus. **c**, Spindle detection probability increases immediately following sync-stimulations (i, *n* = 509 iEEG electrodes) and decreases following mixed-phase stimulation (iii, *n* = 212 iEEG electrodes) relative to sham moments. Black crosses denote the median. *P* = 1.6 × 10^−6^ for sync-stimulation versus sham. *P* = 2.44 × 10^−10^ for mixed-phase stimulation versus sham via Wilcoxon signed-rank tests. *P* = 2.66 × 10^−14^ for sync versus mixed-phase stimulation via Wilcoxon rank-sum test. (ii) Individual memory accuracy enhancement by intervention (*y* axis, as in Fig. 1g) correlates with immediate spindle enhancement (Spearman correlation ρ = 0.69, *P* = 0.013, *n* = 12 participants). Spindle enhancement distribution across all iEEG contacts is shown for each participant; colors as in Fig. 1d. Black crosses indicate the median per participant. Black solid or dashed lines show the linear fit for all participants or sync-stim participants alone, respectively. **d**, Top: Prolonged stimulation-driven change in spindle rate in the 1 min following STIM blocks (post-stim; yellow) compared to the 1 min at the end of pause blocks (pre-stim; gray). Middle: Prolonged stimulation-driven enhancement scores (Methods) in slow-wave event rate (*y* axis) versus sleep spindle event rate (*x* axis). Each dot depicts an iEEG electrode (*n* = 275, 90 and 175 iEEG contacts for red, brown and gray groups, respectively; colors as in Fig. 1c). Spindle rates are elevated in sync-stimulation participants but not in mixed-phase participants. Statistical comparisons via Wilcoxon rank-sum test: *P* = 2.98 × 10^−9^ for slow waves and *P* = 1.42 × 10^–10^ for spindle indices. Whiskers depict the 25th–75th percentiles for sync-stim (red; all stim locations) and mixed-phase contacts (gray). Bottom: Prolonged enhancement of spindle rate following sync-stimulation blocks is widespread across both cortical hemispheres. Each circle (*n* = 275) marks an iEEG electrode in sync-stimulation participants. Circle color represents spindle event enhancement score (Methods); contact location is overlaid on a standard MNI brain template. Source data

**Extended Data Fig. 5:**
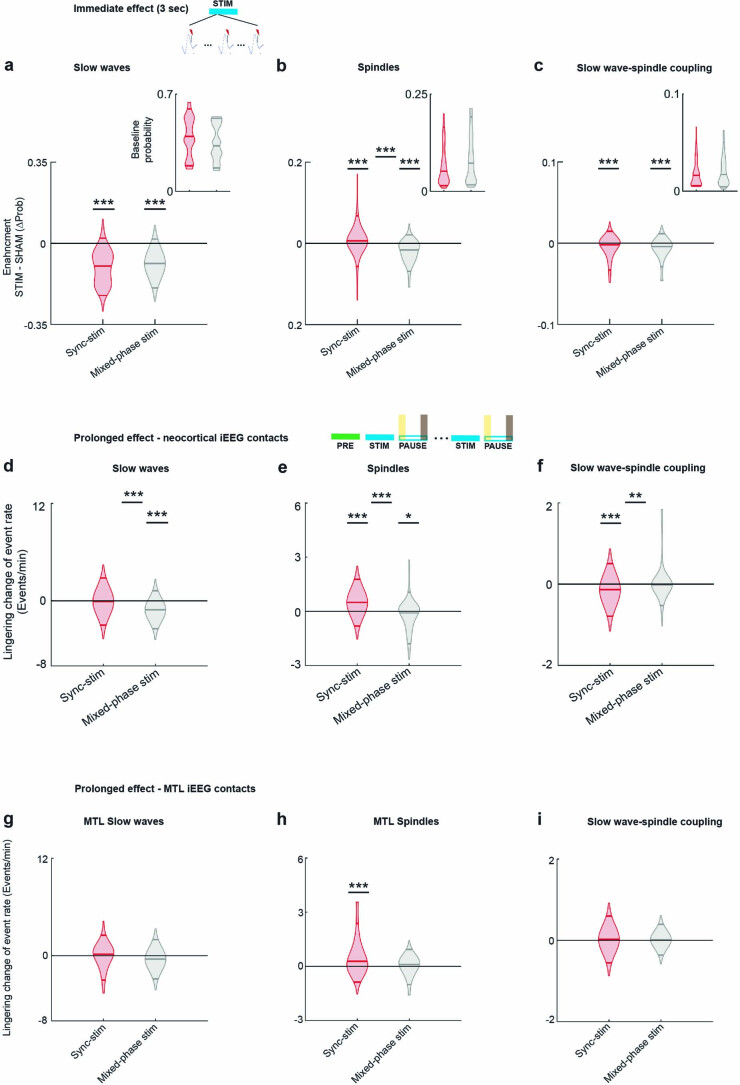
Brain-wide change in sleep oscillation rates following sync-stimulation. **(a-c)** Distributions of immediate change of detection probabilities for slow waves (**a**), spindles (**b**), and slow wave-spindle couples (**c**) for contacts across the brain. Probability was calculated in 3 sec intervals immediately following stimulations, relative to SHAM-stimulation control points (as in Fig. 2c). These distributions reveal decreased probability of slow waves in participants from both stimulation groups (red: sync stim; gray: mixed-phase stim). Wilcoxon signed rank tests are reported for each distribution and rank sum for comparing both distributions: **(a)** slow-waves: P = 8*10^−80^ (sync), P = 3*10^−32^ (mixed), P = 0.08 (between groups), n = 556/215 iEEG contacts for red/gray groups. **(b)** Spindles: P = 6*10^−8^ (sync), P = 4*10^−17^ (mixed), P = 3*10^−23^ (between groups), n = 508/212 iEEG contacts for red/gray groups. **(c)** Slow wave-spindle couples: P = 1.9*10^−5^ (sync), P = 5*10^−8^ (mixed), P = 0.056 (between groups), n = 333/169 iEEG contacts for red/gray groups. n-values differ between panels (a–c) because channels with zero detections in one of the conditions were excluded. Sub-panels depict the distribution of baseline probabilities which are not significantly different between the two stimulation-mode groups. **(d–i)** Distributions of prolonged changes of detection rate for slow-waves, spindles, and slow wave-spindle couples for channels outside the MTL (**d**–**f**) and MTL channels (**g**–**i**). Event rates were calculated over 1-min following stimulations-blocks (yellow shade in top illustration), relative to an equal time range at the end of each ‘pause’ block (gray shade in top illustration). Panels display the difference between rates (events/min). These distributions reveal an increase in spindle event rate in iEEG contacts in the sync-stimulation condition that decays during ‘pause’ blocks, while mixed-phase stimulation contacts exhibit either no change or reduced rates immediately after stim blocks. Wilcoxon signed rank tests are reported for each distribution and rank sum for comparing both distributions: **(d)** slow-waves: P = 0.4 (sync) P = 2*10^−15^ (mixed), P = 4.6*10^−10^ (between groups), n = 269/161 iEEG contacts for red/gray groups; **(e)** Spindles: P = 2*10^−14^ (sync) P = 0.017 (mixed), P = 1.2*10^−12^ (between groups), n = 208/157 iEEG contacts for red/gray groups; **(f)** Slow-wave – spindle couples: P = 1.72*10^−4^ (sync), P = 0.3 (mixed), P = 0.003 (between groups), n = 135/110 iEEG contacts for red/gray groups. N-values differ between panels (d-f) because channels with zero detection in one of the conditions were excluded. (**g)** slow-waves: P = 0.29 (sync) P = 0.053 (mixed), P = 0.052 (between groups), n = 167/54 iEEG contacts for red/gray groups; **(h)** Spindles: P = 9*10^−5^ (sync) P = 0.27 (mixed), P = 0.086 (between groups), n = 106/47 iEEG contacts for red/gray groups; **(i)** Slow wave-spindle couples: P = 0.71 (sync) P = 0.70 (mixed), P = 0.99 (between groups), n = 60/29 iEEG contacts for red/gray groups. N-values differ between panels (g-i) because channels with zero detection in one of the conditions were excluded. *** is used for P < 0.001, * for P < 0.05. Source data

To test whether changes in sleep electrophysiology underlie the observed behavioral changes, we first examined how stimulation modulates slow waves and spindles, known to be tightly linked to memory consolidation^21,39–42^. We used two complementary analysis approaches, in the power domain and in the time domain. In both analyses, a within-session approach compared the modulation in every iEEG contact to its session-specific baseline (see Methods for baseline selection). We evaluated any measure of interest separately for sync-stimulation or mixed-phase stimulation modes (Methods) to test how the temporal accuracy of stimulation affects sleep electrophysiology. First, spectral analysis was used to test whether time–frequency representations (spectrograms) of iEEG signals in multiple brain areas reveal changes in spindle power in an a priori-defined frequency range of 9–16 Hz following stimulation events (Methods). We used a protocol in which short stimulation bursts (50 ms) were delivered, spaced by more than 4 s, during 5-min stimulation blocks interleaved with 5-min pause blocks, during which no stimulation was provided (Methods and Fig. 1b). We first studied the immediate effects of stimulations (as observed within stimulation blocks), during 3-s periods following single stimulation bursts. Sync-stimulation bursts immediately increased sigma (spindle) power relative to a 1-s pre-stimulation baseline across the brain (Fig. 2a and Methods). Importantly, this increase went above and beyond the expected tendency of spindle power to increase around slow-wave active states^35,43^ because it was significantly greater than that found in sham-stimulation moments during intermittent ‘pause’ blocks that had identical delays from MTL slow-wave peaks (Methods; Fig. 2a(ii): Wilcoxon signed-rank test found a significant increase in spindle-band for sync-stimulation contacts; *n* = 565 iEEG contacts from MTL and neocortical sites, *P* < 10^−30^). Conversely, we did not find any immediate change in spindle power in the mixed-phase stimulation group (Wilcoxon signed-rank test, *n* = 215, *P* = 0.73). Nor did we find an increase in post-stimulation power when examining a control frequency range (20–27 Hz). Next, we compared the immediate effects of sync-stimulation and mixed-phase stimulation modes on slow waves and spindles in the time domain, by selectively identifying individual slow-wave and spindle events on each iEEG contact, according to established detection algorithms^35,44^ (Fig. 2b and Methods). The probability of slow-wave events was reduced for both sync-stimulation and mixed-phase conditions immediately following stimulation (Extended Data Fig. 5a). However, a significant increase in spindle detection probability was observed immediately following sync-stimulation (in the 3-s inter-stimulus interval) compared to sham-stimulation time points with identical delays from MTL slow-wave peaks (Fig. 2c(i); Wilcoxon signed-rank test, *P* < 10^−4^). Conversely, in the mixed-phase condition, immediate iEEG spindle detection probability was significantly decreased (Fig. 2c(ii); *P* < 10^−8^, Extended Data Fig. 5b). Critically, when considering all participants (both sync-stimulation and mixed-phase stimulation), behavioral changes in recognition memory accuracy were highly correlated with the degree to which stimulation affected immediate spindle occurrence (Fig. 2c(iii); Spearman correlation ρ = 0.69, *P* = 0.013; *n* = 12 participants). A high correlation coefficient between immediate spindle increase and memory increase was also observed when focusing on the smaller group of nine participants receiving sync-stimulation, but this did not reach statistical significance (Fig. 2c(iii); ρ = 0.53, *P* = 0.148).

Next, we also tested for prolonged effects of stimulation occurring beyond the 5-min stimulation blocks, by comparing the rates of iEEG sleep oscillations in the 1-min following each stimulation block with the 1 min at the end of each ‘pause’ block, using a normalized pre/post index (Methods). We found that sync-stimulation led to prolonged enhancement of spindle rate, whereas mixed-phase stimulation led to prolonged decrease in spindle rate (Fig. 2d and Extended Data Fig. 5e; Wilcoxon rank-sum test comparing sync-stim and mixed-phase spindle enhancement index distributions; *P* < 10^−8^). Interestingly, prolonged spindle rate increase was observed in both hemispheres across wide cortical territories including MTL and neocortical electrodes (Fig. 2d and Extended Data Fig. 5d–i; Wilcoxon rank-sum test comparing neocortical and MTL spindle enhancement index distributions, *P* = 0.13; Methods). While slow-wave rates following sync-stimulation were comparable before and after stimulation blocks, mixed-phase stimulation led to a significant reduction in slow-wave occurrence when assessing prolonged effects (Fig. 2d and Extended Data Fig. 5d; Wilcoxon rank-sum test comparing sync-stim and mixed-phase slow-wave enhancement index distributions, *P* < 10^−7^). Together, analysis of slow waves and sleep spindles revealed that deep brain stimulation (DBS) that is synchronized to MTL slow-wave active phases leads to robust and widespread effects that persist beyond stimulation blocks, particularly enhancing spindles in a manner correlated with memory benefits.

### Stimulation increased spike phase-locking to medial temporal lobe slow waves

**Fig. 3:**
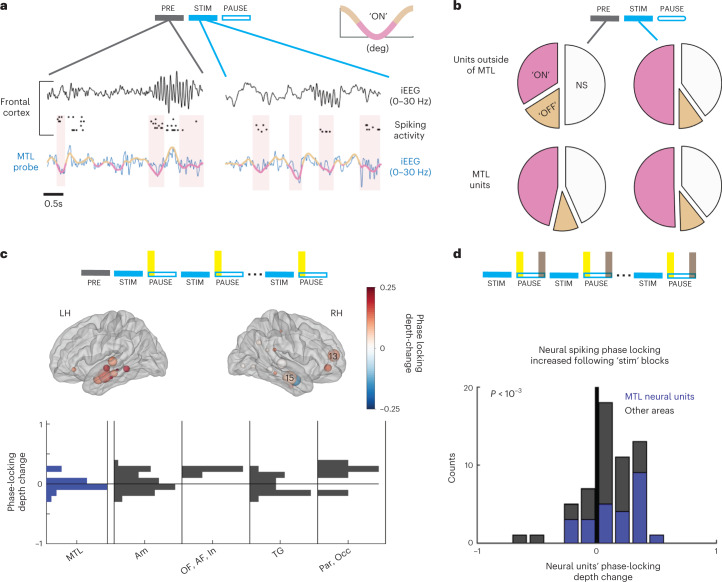
Neuronal spiking across the brain phase locks to medial temporal lobe slow-wave activity following synchronized stimulation. **a**, Two representative examples show 4 s of orbitofrontal cortex spiking activity during sleep before (left) and during (right) stimulation. Rows (top to bottom) show prefrontal iEEG (black, filtered 0–30 Hz), spiking in four neuronal clusters (black ticks) and MTL probe iEEG (blue) superimposed with slow-wave active (pink) versus inactive (brown) phases. Spiking activity before stimulation is scattered and becomes phase locked to MTL active phase (pink) during stimulation blocks. iEEGs were *z*-scored for visualization. **b**, Analysis across neuronal population: Fraction of units showing significant phase locking to MTL ‘ON’ phase (pink), to MTL ‘OFF’ phase (brown) or no significant (NS) phase locking (white). Top row, outside MTL (*n* = 190), the percentage of locked clusters increased from 34.0% during baseline (‘PRE’, gray block, left pie chart) to 50.0% during stimulation block (‘STIM’, blue, right pie chart). In MTL, the percentage of phase-locked units (*n* = 107) remained stable (46.5% pre-stim (left) and 50.5% during stim block (right)). **c**, Prolonged increase (1 min after stimulation, yellow intervals) in phase locking to MTL slow waves (quantified by locking depth change; Methods) is widespread across cortex in both hemispheres regardless of stimulation location. Each circle shows the anatomical location of neuronal clusters overlaid on a standard (MNI) brain template. Circle color represents changes in phase locking for that region (color bar on right). Circle size reflects the number of units detected in that region (largest spheres have numbers overlaid). Bottom, locking depth change distributions by regions. MTL includes hippocampus, entorhinal cortex and parahippocampal gyrus. Am, amygdala; OF, orbitofrontal cortex; AF, anterior prefrontal cortex; In, insula; TG, temporal gyrus; Par, parietal cortex; Occ, occipital cortex. **d**, Prolonged neural phase-locking increase (Methods, time periods as in Fig. 2d, yellow versus gray periods highlighted in top illustration). Locking depth change distributions are stacked for neural units located in MTL (blue) and other areas (dark gray); *n* = 57 units met firing rate criteria; *P* = 5 × 10^−4^ via Wilcoxon signed-rank test. Source data

**Extended Data Fig. 6:**
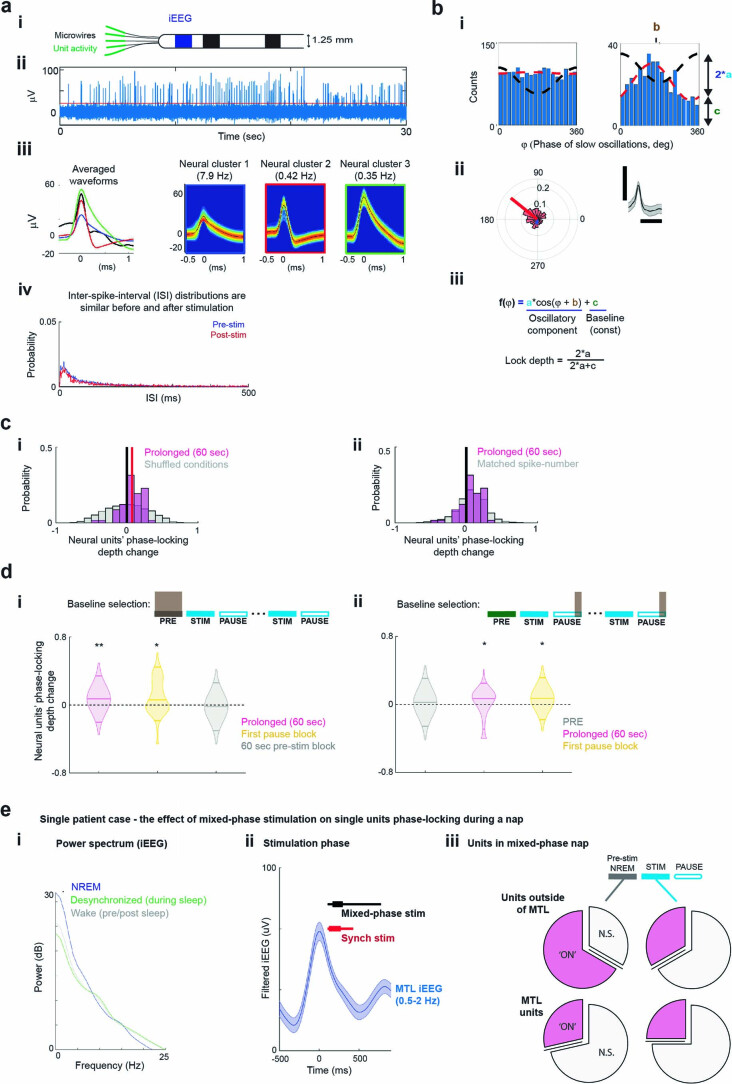
Phase-locking change following stimulation. **(a)** Spike sorting procedure: **(i)** top illustration - flexible depth electrodes used for simultaneous recording of iEEG (platinum contacts, blue and black) and unit spiking activity (recorded on microwires, green). **(ii)** Representative 30-sec example of high-pass filtered (>300 Hz) microwire LFP signal recorded in prefrontal cortex along with threshold for spike detection (red horizontal line). **(iii)** Screenshot from ‘wave clus’ spike-sorting toolbox^91^ demonstrating automatic superparamagnetic clustering of wavelet coefficients for 3 clusters. Left – average waveform for 3 detected clusters, right – each cluster’s waveform (mean and standard deviation) displayed as a heat map^91^. **(iv)** Example: Inter spike interval (ISI) distribution for cluster #1 during pre-stim sleep and post-stim sleep (correlation between distributions is 0.96). **(b) Temporal-fit method for spike phase distribution:** Distribution of spike-phases from a neural unit recorded in orbitofrontal cortex, phases calculated for MTL iEEG slow-wave signal. **(i)** Left - before any stimulation block (‘PRE’), right - during the first ‘pause’ block, demonstrating a prolonged effect of sync-stimulation. Colored letters correspond to fitted values in the equation plotted in bottom panel (see below). **(ii)** Left: same distributions as in (i) in blue (for ‘PRE’), and in red (prolonged condition), overlaid on a polar plot, with mean direction and resultant vector length computed with circstat toolbox^93^ (Matlab, Mathworks); Right - average and SEM of action potential waveform during the entire intervention session. Calibration bars mark 1 msec and 50 µV. **(iii)** Equation used for fitting the phase distribution and quantifying locking: specific elements used for calculating phase-locking depth are color-coded and shown also on the example distribution plotted in top panel: red dashed line, fitted function. a = amplitude/gain, b = preferred phase. c=baseline (mean firing rate/DC). (**c**) **(i)** The distribution of depth-lock change in the prolonged condition (pink, aggregated for n = 65 neural units included in Fig. 3e) is significantly different from a shuffled distribution in which baseline and evaluated condition values are mixed (gray**). (ii)** The distribution of depth-lock change isn’t significantly different from a bootstrapped distribution calculated based on a sub-set of spikes to test for biases due to firing rate changes (Kolmogorov-Smirnov two-sample test, P = 0.42) **(d)** Changes in lock-depth for units outside of MTL are not dependent on selection of baseline. (i) and (ii) compare distributions of change relative to two possible baselines (shown in gray shade on each timeline): the PRE-block before any stimulation (i) or aggregated 1-min periods preceding all stim blocks (ii). Each violin plot depicts a distribution of phase-lock changes for a different condition. Pink: prolonged effect (1-min following stim blocks), Yellow: first pause block, Gray: the other panel’s baseline (gray). (i). There is an increase in phase locking for the prolonged time point relative to the baseline (pink; n = 47 units; P = 0.008), as well as during the entire first pause block (yellow; n = 27, P = 0.04). (ii) Baseline: gray; n = 35, P = 0.49. Prolonged: pink; n = 32, P = 0.024. First pause block: yellow, n = 25, P = 0.028. P-values are based on Wilcoxon rank-sum test. Note that inclusion criteria result in slightly different population sizes for each pair of conditions but results are consistent with Fig. 3d, e for all condition-pairs. **(e**) **Phase locking changes following mixed phase stimulation**. Reporting the results of a single stimulation session recorded during a daytime nap. The session included interleaved synched and mixed-phase stimulation blocks. We did not find elevated phase-locking of single units for this session (n = 14 single units; 6 from MTL) **(i)** iEEG power spectrum for each vigilance state for nap session. Blue, NREM sleep. Green, desynchronized states (REM sleep or sporadic wake intervals). Gray, unequivocal wakefulness periods occurring before and after the daytime sleep session. **(ii)** Average and SEM of MTL probe’s iEEG signal (blue trace, filtered between [0.5–4]Hz to highlight slow-wave activity), time-locked to the positive iEEG peak immediately preceding stimulation time (t = 0). Box-plots depict the distribution of stimulation delays iEEG slow-wave positive peak for this participant – red is sync-stim blocks and black is mixed-phase stim blocks. The bounds of the boxes represent the interquartile range and whiskers extend between 1–99 percentiles. **(iii)** Comparison of units’ phase-locking to MTL iEEG pre-stim (left pie charts) and during stimulation blocks: While units in the MTL were not affected by mixed-phase stimulation, the number of non-significantly phase-locked units outside the MTL increased during stimulation blocks (an opposite trend than we observed in sync-stimulation sessions – see Fig. 3c). Pink – units phase-locked to MTL iEEG ‘ON’ phase (90–270 degrees); White, non-significant phase-locking (we did not observe units phase-locked to ‘OFF’ phase in this session). Source data

Next, we assessed the effects of sync-stimulation on phase locking of spiking in neural units to MTL. We quantified how sync-stimulation affected the timing of neuronal action potential discharges recorded on individual microwires (Extended Data Fig. 6a(i)) across multiple brain regions, with respect to MTL iEEG slow-wave phase (0.5–4 Hz; this signal was used for stimulation timing; Supplementary Table 4). For each neuronal unit (spike-sorted offline; Methods), we calculated the phase of each spike relative to the MTL slow wave and fitted each distribution with a cosine function to evaluate the depth of phase locking and preferred firing phase (Methods and Extended Data Fig. 6b; *n* = 325 neuronal units in eight sync-stimulation participants with microwire recordings). First, we evaluated the changes in neuronal firing by comparing phase-locking depth during stimulation blocks (apart from intervals around stimulation bursts) and pause blocks to several baseline options (Methods, Extended Data Fig. 6c,d and Fig. 3a). To include as many action potential events in this analysis—even for low firing rate units—we focused on long intervals, combining all 5-min stimulation blocks (‘stim’) and comparing them with 10–15 min periods of baseline activity before the first stimulation block (‘PRE’). We found that sync-stimulation increased the proportion of neuronal units outside the MTL with significant phase locking to MTL iEEG active states from 34% to 50% (Fig. 3b), but this percentage was only modestly altered for MTL units (46.5% to 50.5%; Fig. 3b). Next, as examined for sleep spindles, we investigated potential prolonged effects in the 1-min intervals beyond stimulation blocks to better understand the dynamics and regional variability of phase-locking changes (Methods). In the first minute following every stimulation block, the percentage of phase-locked cells returned to baseline (34% for units outside MTL) but units increased the phase-locking depth relative to baseline, and this was specific to neural units outside the MTL where observed effects were significant (Fig. 3c, Wilcoxon signed-rank test; units outside the MTL: *P* = 0.007/*n* = 47; units in the MTL: *P* = 0.9/*n* = 26, but Wilcoxon rank-sum test comparing populations *P* = 0.1). Even when restricting the data to compare only the first minute following each stimulation block with the last minute of the previous pause block, we found significantly higher depth of phase locking immediately after sync-stimulation (Fig. 3d; Wilcoxon signed-rank test when aggregating all neural units—*P* < 10^−3^). This result represents a prolonged effect with dynamics similar to the decay profile we observed for spindles and slow waves, decreasing within minutes. Prolonged effects were validated via two shuffling procedures (Methods)—by verifying that real data significantly differed from a shuffled distribution with randomly assigned condition labels (Extended Data Fig. 6c; Wilcoxon rank-sum test, *n* = 57 neural units, *P* = 0.02), and by verifying that phase locking is not dependent on possible changes in mean firing rates (Extended Data Fig. 6c; Kolmogorov–Smirnov two-sample test, *P* = 0.7). The presence of robust prolonged effects allowed us to confidently interpret changes as true differences in synchronization rather than potential contamination by stimulation artifacts. Together, we found that sync-stimulation led to prolonged increases in phase locking of neuronal spiking activity, particularly in distant regions, to MTL slow waves, which decayed within minutes.

### Increased ripple-slow wave-spindle coupling correlates with memory

**Fig. 4:**
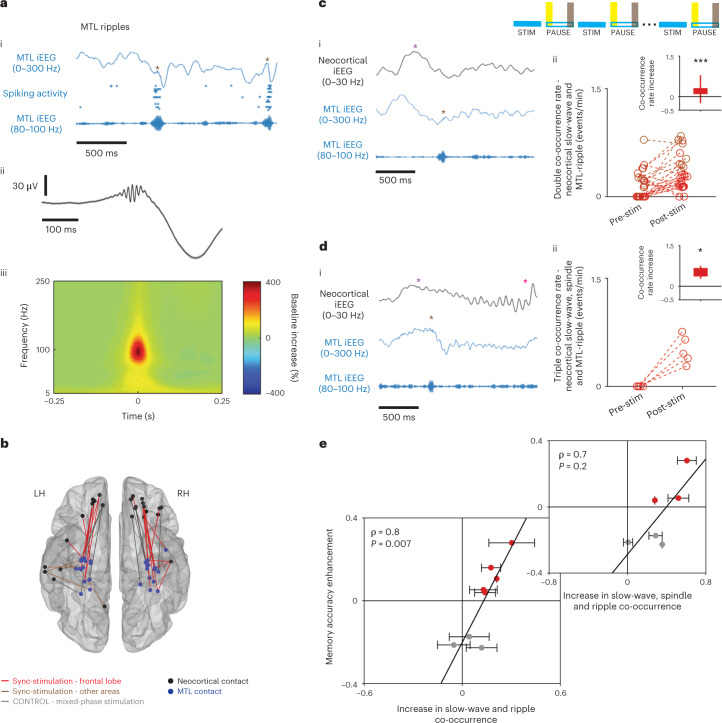
Synchronized stimulation increases triple co-occurrence of medial temporal lobe ripples, neocortical slow waves and thalamocortical spindles. **a**, (i) Example ripples (brown asterisks) detected in MTL (parahippocampal gyrus) iEEG: top and bottom rows show iEEG signal filtered (0–300 Hz or 80–100 Hz, respectively). Middle row, spiking activity on adjacent microelectrodes. (ii) Grand average of unfiltered iEEG aligned to the maximum ripple peak (mean ± s.e.m., *n* = 7,172 ripple detections in 28/13 iEEG channels/participants during pre-stim epochs). (iii) Average ripple-peak-locked TFR (percentage change from 1-s baseline) highlights the band-limited frequency profile of detected ripples**. b**, All pairs of neocortical (black) and MTL (blue) iEEG electrodes used in subsequent co-occurrence analysis, overlaid on a standard (MNI) brain template (*n* = 41 iEEG electrode pairs, 15 participants). Line colors as in Fig. 1d. **c**, Double co-occurrence of MTL ripples and neocortical slow waves: (i) Example: simultaneous recording of neocortical slow wave (top; purple asterisk shows positive iEEG peak) and MTL ripple (middle and bottom; brown asterisk shows detected ripple). (ii) Incidence of double co-occurrence significantly increased in the 1-min interval post-stimulation blocks (yellow) relative to 1-min end of pause block (gray). Inset, box plot of differences between post-stim and pre-stim incidence rates (*n* = 25 electrode pairs in 10 participants in sync-stimulation group; *P* = 8.2 × 10^−4^, right-tailed Wilcoxon signed-rank test). Line colors as in Fig. 1d. See Extended Data Fig. 9d for mixed-phase distribution. **d**, Triple co-occurrence of MTL ripples, neocortical slow waves and thalamocortical spindles: (i) Example signals as in **c**. Pink asterisk denotes spindle identified shortly after slow-wave/ripple event. (ii) Incidence of triple co-occurrence significantly increased in the 1-min interval post-stimulation blocks (yellow) relative to 1-min end of interval block (gray). Inset, box plot of differences between post-stim and pre-stim incidence rates (*n* = 5 electrode pairs in 3 participants, *P* = 0.03, right-tailed Wilcoxon signed-rank test). See Extended Data Fig. 9e for mixed-phase distribution. Box plots in panels **c** and **d** represent interquartile range, whiskers mark the 1–99 percentiles. **e**, Memory accuracy enhancement following intervention (*y* axis) correlates with increase in MTL ripples-neocortical slow waves double co-occurrence (*x* axis; Spearman correlation, ρ = 0.8, *P* = 0.007; *n* = 30 MTL–neocortical electrode pairs in 8 participants). Markers are median values per participant, bars are the s.e.m.; colors as in Fig. 1d; black line shows linear fit based on median values per participant. Subpanel shows correlation between memory accuracy enhancement and stringent triple co-occurrence criteria (MTL ripples, neocortical slow waves and spindles; *n* = 12 pairs, 6 participants, ρ = 0.7, *P* = 0.2). iEEGs were *z*-scored for visualization. Source data

**Extended Data Fig. 7:**
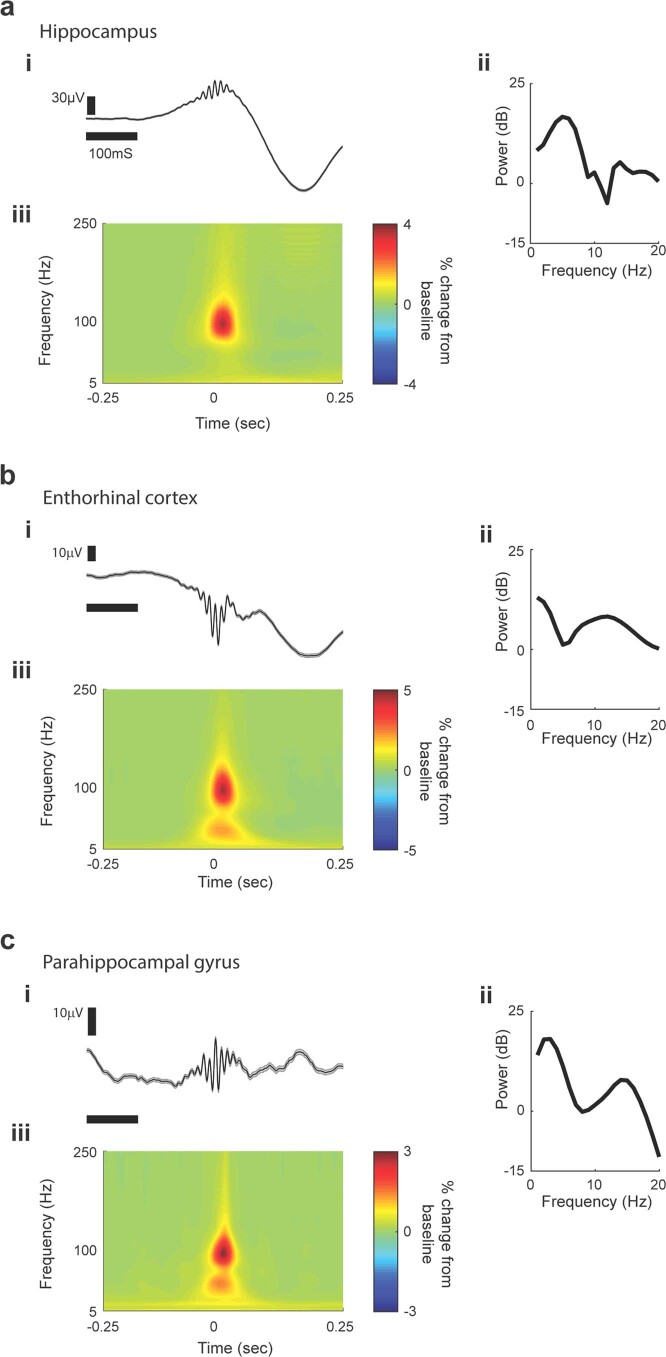
Ripple characteristics in specific MTL regions. **(a)** Detected ripples in iEEG electrodes targeting hippocampus (i) Grand average of raw unfiltered iEEG traces (n = 3685 detected ripple events in 12 electrodes/10 patients, mean ± s.e.m.) aligned to the maximum of the ripple peak during pre-stim epochs. (ii) Average power spectrum of iEEG traces (±1 sec around detected ripples). (iii) Average of ripple-peak-locked TFR (time-frequency representation, % change from pre-event baseline, color bar on right) highlights the band-limited nature of ripples around 80–120 Hz. **(b)** Same format as panel a for detected ripples in iEEG traces of electrodes targeting entorhinal cortex (n = 2646 events in 10 electrodes/7 patients). **(c)** Same format as panel a for detected ripples in iEEG traces of electrodes targeting parahippocampal cortex (n = 841 events in 6 electrodes/5 patients). Power spectrum reveals peaks at ~3 Hz and ~14 Hz (fast sleep spindles). Calibration bars mark 100 ms and 30μV (hippocampus) or 10μV (other MTL regions). Source data

**Extended Data Fig. 8:**
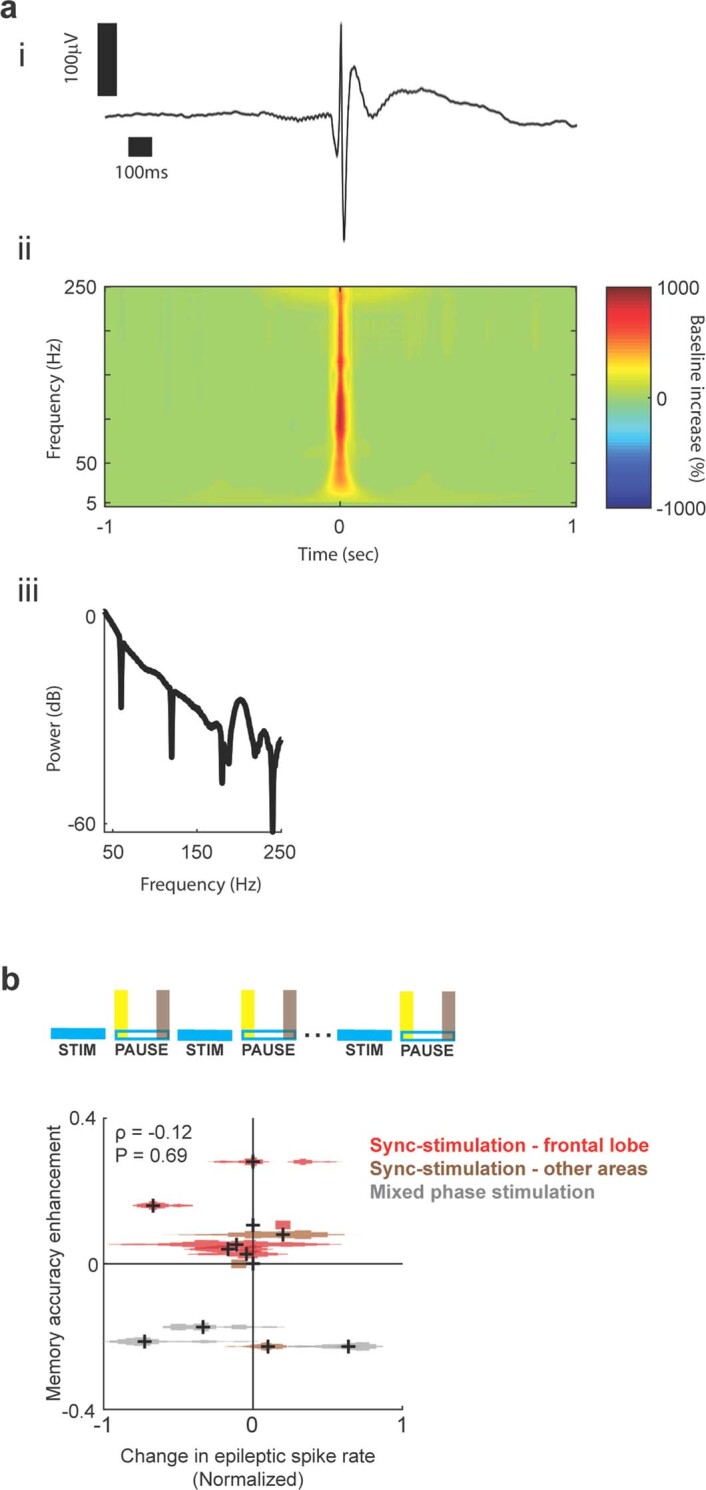
Pathological interictal epileptiform discharges (IEDs). **(a) (i)** Grand average of 5819 unfiltered iEEG traces during pre-stim intervals (mean ± s.e.m) in 33 electrodes with prevalent IED activity based on visual review and neurologist definition (n = 7 participants), aligned to the maximum IED peak (time 0). Note that these channels were excluded from main analyses based on high rate of abnormal activity. Calibration bars mark 100 ms (x-axis) and 100μV (y-axis). (ii) Average of IED-locked TFR (% change from pre-event baseline, color bar on right), highlighting the wide-band and high-frequency spectral profile of IEDs. (iii) Grand average iEEG power spectrum around (±1 s) detected IED events (1–300 Hz, **1** Hz resolution). (**b**) Effects of stimulation on overnight memory accuracy enhancement (y-axis) vs. change in IED rates (x-axis) do not reveal a consistent relationship: Recognition memory accuracy enhancement per subject (values as in Fig. 1g) vs. median value of each participant’s distribution of IED change does not show a significant correlation (Spearman correlation: ρ = −0.12, P = 0.69, n = 12 participants). The distribution for each patient across all iEEG contacts is shown; IED event rates were calculated over 1-min following stimulations-blocks (yellow shade in top illustration), and normalized relative to an equal time range at the end of each ‘pause’ block (gray shade in top illustration). Color corresponds to stimulation type, as in Extended Data Fig. 3; black crosses mark the median value for each patient. Source data

Next, we focused on the coordination between hippocampal ripples and thalamocortical slow waves and spindles, previously suggested to mediate sleep-dependent memory consolidation. To this end, we focused on a subset of 16 participants where hippocampal and prefrontal activities were simultaneously monitored. Ripples (Fig. 4a; *n* = 7,172 events) were detected in MTL iEEG electrodes during pre-stimulation sleep using an automated algorithm^44^ (Methods), using bipolar referencing to minimize volume conduction effects. Most events were detected in the hippocampus (CA1, CA3/DG and subiculum), but also in adjacent entorhinal cortex and in the parahippocampal gyrus (see Extended Data Fig. 7 for breakdown to different MTL sub-areas), where ripples have been previously reported^28,45–47^. An extensive inspection of detected ripple events, along with their narrow-band frequency profile around 80–120 Hz, attests to largely successful separation from pathological high-frequency MTL oscillations and interictal epileptiform discharges (IEDs), which exhibited a distinct spectral profile with wide-band higher-frequency pathological activity (Extended Data Fig. 8a).

**Extended Data Fig. 9:**
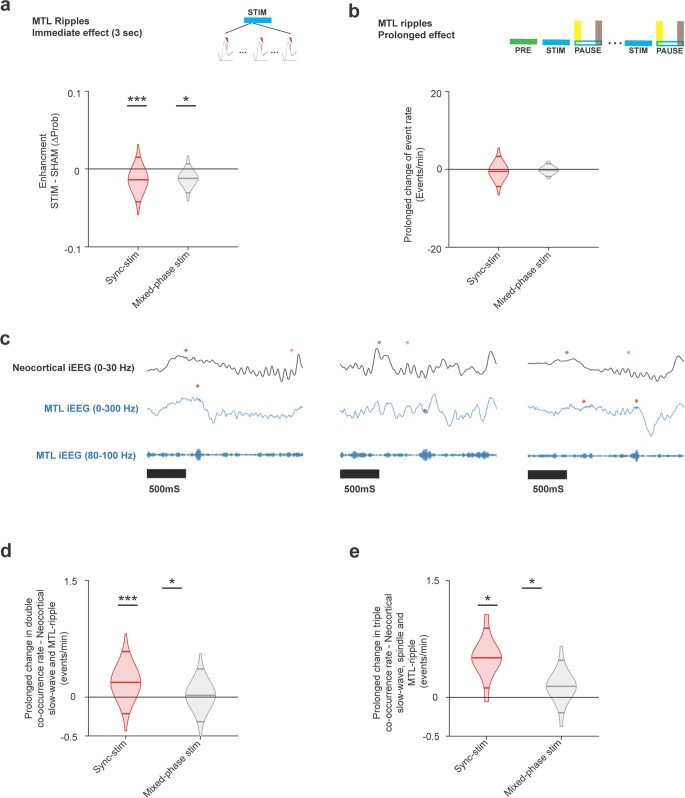
Coupling of sleep oscillation between MTL and neocortex. **(a)** Immediate effect of stimulation on ripple detection. We found a significant reduction, in both stimulation protocols, in ripple event detection probability on MTL iEEG electrodes calculated during 200 ms following stimulation bursts, relative to sham stimulation points. P-values are reported for a Wilcoxon sign-rank test for each distribution. Red: MTL contacts in sync-stimulation patients (n = 18 iEEG contacts, P = 0.004); gray: MTL contacts in mixed-phase stimulation patients (n = 8 iEEG contacts, P = 0.01). Only MTL contacts ipsilateral to the closed-loop input (probe) are included. No significant difference was found between distributions (rank-sum Wilcoxon test. P = 0.7). Note that stimulation was delivered in neocortical sites, distant from MTL (Fig. 1d). **(b)** Prolonged effect of stimulation on ripple detection: Event rates were calculated over 1-min following stimulation blocks (yellow shade in top illustration), relative to an equal time range at the end of each ‘pause’ block (gray shade). Ripple detection rates were stable in MTL channels during ‘pause’ blocks. Colors as in (**a**). Wilcoxon sign-rank test for each distribution: Red: n = 18, P = 0.2; Gray: n = 8, P = 0.7. **(c)** Examples of triple co-occurrences of neocortical slow-waves, thalamo-cortical-spindles and MTL-ripple events: each example displays simultaneous recordings from a pair of iEEG electrodes in neocortex (black, top row, 0–30 Hz), MTL (blue, middle row, 0–300 Hz) and a ripple band (80–100 Hz) band-pass filtered trace of the MTL iEEG (bottom row). Brown star, detected ripple; Purple, detected slow-wave positive iEEG peak (‘OFF’ period), pink – detected spindle event. Calibration bars mark 500 ms (x-axis), for visualization purposes iEEG data were z-scored over a 2-sec period plotted in panel. Examples from participants 2 and 14 (**d**) Prolonged change in MTL ripple-neocortical slow wave co-occurrence incidence: distribution of differences between post-stim period vs pre-stim period is plotted for iEEG channel couples from each stimulation-mode group. Wilcoxon right-tail signed-rank test: Red: sync-stim, n = 25 iEEG couples, P = 0.0008; gray: mixed-stim, n = 13 iEEG couples, P = 0.36. Wilcoxon rank-sum test between groups: P = 0.049). (**e**) Prolonged change in MTL ripple-neocortical slow wave, thalamo-cortical spindle triple co-occurrence incidence: distribution of differences between post-stim period vs pre-stim period is shown for iEEG channel couples from each stimulation-mode groups. Wilcoxon right-tail signed-rank test: Red: n = 5 sync-stim iEEG couples, P = 0.031; Gray, n = 7 mixed-stim iEEG couples, P = 0.054, Wilcoxon rank-sum test between groups: P = 0.01). N-values differ between panels (d, e) because channels with zero detection in both conditions were excluded. *** is used for P < 0.001, * for P < 0.05. Source data

Ripple detection probability decreased immediately following stimulation bursts (in 3-s inter-stimulus intervals) relative to sham moments in stimulation-free intervals, in both sync-stimulation and mixed-phase stimulation (Extended Data Fig. 9a and Methods; Wilcoxon signed-rank test: *P* = 0.004/*n* = 18 and *P* = 0.0.01/*n* = 8 for sync-stim and mixed-phase stim iEEG contacts, respectively, no significant difference between groups). However, this reduction did not extend beyond stimulation blocks, as we did not find a prolonged change in ripple rate when focusing on the first 1-min intervals of pause periods as in other analyses (Methods and Extended Data Fig. 9b; Wilcoxon signed-rank test yielded nonsignificant *P* values for both stimulation modes and no significant difference was found between groups).

**Extended Data Fig. 10:**
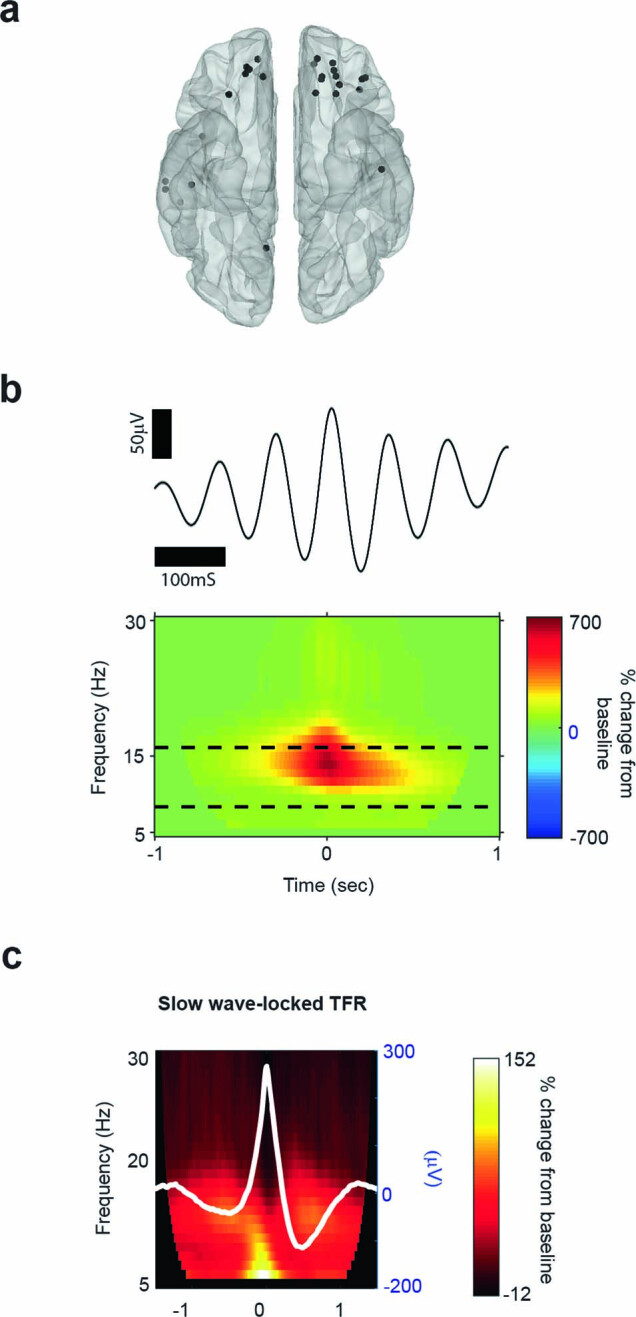
High frequency sleep spindles detected in cortical channels. **(a)** Neocortical iEEG contacts included in triple-coupling analysis between sleep oscillations in neo-cortex and MTL ripples (n = 41, black), overlaid on a standard (Montreal Neurological Institute) brain template. **(b)** High-frequency spindles (above 11 Hz), in iEEG electrodes included in triple-coupling analysis. Top: Grand average of raw unfiltered iEEG traces (n = 3764 events in 41 electrodes from11 patients, mean ± s.e.m) aligned to the maximum of the spindle peak during PRE-stim epochs; bottom: average of spindle-peak-locked TFR (time-frequency representation, % change from pre-event baseline, color bar on right) highlights the band-limited nature of spindles around 9–16 Hz (marked by dashed lines). **(c)** Average of slow wave-peak-locked TFR highlights the increase in spindle-frequency-band (9–15) Hz around slow-wave troughs calculated for slow-waves detected in PRE-stim blocks (same iEEG contacts as in panels a, b). Mean± s.e.m slow wave trace is superimposed in white (scale shown on righthand y-axis). Source data

Although we did not find an increase in the incidence of ripple events, we asked whether synchronizing stimulation induced an increase in temporal coupling between hippocampal ripples and oscillations outside the MTL, namely neocortical slow waves and thalamocortical spindles. We evaluated the coupling incidence in cross-brain electrode pairs such that one electrode was placed in MTL and exhibited ripples during pre-stimulation sleep and the other was located in the neocortex with robust spindle activity (all neocortical contacts except five were located in the orbitofrontal cortex; Fig. 4b and Methods). We further focused on fast (>11 Hz) sleep spindles (Methods and Extended Data Fig. 10), because these are preferentially associated with memory consolidation^42,48^, are more synchronized with slow-wave active phases^48^, and are associated with hippocampus activation^49^, even though they are not as prevalent in prefrontal cortex^35^.

We found that sync-stimulation, but not mixed-phase stimulation, enhanced the temporal coupling between MTL ripples and neocortical slow waves in the 1-min following every stimulation block, relative to 1 min preceding stimulation blocks (prolonged effects as in other electrophysiology analyses; Methods and Fig. 4c, right-tailed Wilcoxon sign-rank test: **P < 10^−3^, *n* = 25 pairs, for sync-stimulation; *P* > 0.5, *n* = 13 pairs for mixed-phase stimulation; rank-sum Wilcoxon test comparing distributions of changes in both stimulation modes, *P* < 0.05; Extended Data Fig. 9d). Furthermore, using a more stringent triple co-occurrence criterion of cortical slow waves, thalamocortical sleep spindles and MTL ripples (Methods), we also found that sync-stimulation increased co-occurrence of hippocampal and thalamocortical sleep oscillations (Fig. 4d; right-tailed Wilcoxon sign-rank test: *P = 0.03/*n* = 5 and *P* > 0.5/*n* = 7, for sync-stimulation and mixed-stimulation groups, respectively, rank-sum Wilcoxon test comparing distributions of changes in both stimulation modes: *P* = 0.01; Extended Data Fig. 9e).

Finally, to test our initial hypothesis, that is, whether increased co-occurrence of hippocampal and thalamocortical sleep oscillations mediates overnight memory consolidation, we examined the relationship between electrophysiology and memory (Fig. 4e). We observed a robust correlation between recognition memory accuracy change and increase in co-occurrence of sleep oscillations—MTL ripples and neocortical slow waves (*n* = 30 MTL-cortical electrode pairs in eight participants; Spearman correlation calculated for all pairs ρ = 0.8, *P* = 0.007; Methods). When focusing only on smaller subsets of data (for example, only sync-stimulation data, or triple co-occurrence of ripples, spindles and slow waves; Fig. 4e), we observed positive correlations that did not reach significance (ρ = 0.8, *P* = 0.1, *n* = 18 pairs; and ρ = 0.7, *P* = 0.2, *n* = 12 pairs, respectively). Together, our results support the notion that improving the temporal coupling between human MTL ripples and thalamocortical sleep oscillations is key for overnight memory consolidation.

## Discussion

Dynamically modulating the coupling between MTL ripples, cortical slow waves and thalamocortical spindles in human sleep via intracranial DBS synchronized in real time with MTL slow-wave active periods reveals a robust relation between MTL–neocortical coupling and overnight consolidation of recognition memory. In addition, we show that sync-stimulation enhances spindle activity and improves phase locking of brain-wide neuronal spiking activity to MTL slow waves. Importantly, across all participants receiving either sync-stimulation or mixed-phase stimulation, individual overnight changes in memory accuracy are tightly correlated with these electrophysiological effects. The efficacy of the closed-loop intervention was observed when applying temporally precise stimulation relative to local MTL activity—made possible by its real-time monitoring—but not with stimulation that was not precisely timed.

Our data demonstrate that multiple neocortical areas distant from the stimulation focus, even in the contralateral hemisphere, undergo similar coupling to the MTL following prefrontal sync-stimulation, as does the prefrontal cortex adjacent to the stimulation site. Wide-brain prolonged effects are likely made possible by the unique state of brain activity during sleep that allows local, low-amplitude intracranial stimulation to propagate effectively across wide cortical territories^50^. While we establish that prefrontal white-matter DBS leads to robust electrophysiological and memory effects, stimulation in other neocortical sites may also lead to similar effects. Nevertheless, the effects observed with our stimulation sites are consistent both with the efficacy of white-matter stimulation^37,51–54^ and with the known role of MTL–prefrontal interactions in memory^20,22,33,34^.

Paired-associate learning (PAL) paradigms have been widely used in studies of human sleep and declarative memory ever since the early pioneering studies by Jenkins and Dallenbach^1^. Typically, word-pair associations are used to reveal that sleep is associated with reduced forgetting compared with wake intervals^39,42^. In this study, we used a visual paired-associate learning (vPAL) task^55^ of image associations, using a naturalistic approach suited for clinical settings in which learning took place during a one-shot viewing session. We tested participants on their memory for the associations, as well as introducing ‘lure’ images to test for recognition memory. The same lures were used in evening and morning testing, so the morning test can be described as a source recognition test^56^ requiring participants to distinguish learned images from lures they have seen before but in a different context. With this task, we found that our intervention improved recognition memory accuracy, measured by the difference between correct recollection (hit rate) and false lure acceptance (false alarms; Fig. 1g and Extended Data Fig. 4c), whereas pairing performance was only modestly increased (Extended Data Fig. 4a,b). Our data suggest that the dominant factor in the performance increase associated with sync-stimulation was a reduction in falsely tagging lures (Extended Data Fig. 4e), in line with previous studies that pointed to the effect of sleep on minimizing false memory formation^57–59^. The fact that sleep specifically benefited recognition accuracy in this memory task was also reported in a separate cohort of healthy participants^55^. Several factors may contribute to the difference between our results, where association performance was not significantly impacted, and previous PAL-based studies. Such factors include lower initial memory strength related to the lack of rehearsal during the learning phase, the use of an immediate memory retrieval that may constitute a re-consolidation step, and the type of stimuli used (easily recognizable images of celebrities versus words; see also Discussion in ref. ^55^). Additional studies are needed to determine how sleep affects consolidation of memory accuracy and association.

The participants in the current study were individuals with medically refractory epilepsy. Their pathology and medication can affect sleep in multiple dimensions (reviewed in ref. ^60^). Briefly, refractory temporal lobe epilepsy is associated with excessive daytime sleepiness and changes to sleep architecture^61^ when nocturnal seizures may occur. Anti-epileptic drugs reduce the amount of rapid eye movement (REM) sleep^62^, and changes in the dynamics and power of slow waves and sleep spindles have been observed in this population^61,63^. In addition, IEDs occur preferentially during NREM sleep^64^, whereby elevated neuronal synchrony within thalamocortical networks facilitates the spread of focal IEDs to distant brain areas^65^. While we acknowledge that these aspects represent potential confounding factors, several observations suggest that these issues do not likely play a major role in our findings. First, anticipating variability in participant age, cognitive abilities, epilepsy profile and medication regime (Supplementary Table 2), we designed our cognitive paradigm as a ‘within-participant’ design (rather than a within-group comparison) thereby minimizing the contributions of these factors. Second, we observed similar behavioral and electrophysiology results despite variability in clinical profiles (Supplementary Table 1) and medication regimes (Supplementary Table 2), arguing against a major role. Third, while previous studies suggest that IEDs may impair memory by interfering with physiological hippocampal–cortical coupling^66^, we did not observe an association between recognition memory accuracy and the degree to which stimulation affected IEDs (Methods and Extended Data Fig. 8b), arguing against the possibility that memory benefits reported here are driven by IED reduction. Additional studies are needed to generalize findings based on this participant population to the general population.

Where do our results stand in relation to previous literature on boosting memory via closed-loop manipulations and sleep interventions? In humans, both open-loop and closed-loop intracranial electrical stimulation during the encoding phase have been previously reported in awake participants—by our laboratory and others—to benefit memory performance^51,67–69^. The current study highlights an alternative approach of interventions during offline memory consolidation, where sleep offers a privileged window of opportunity^70^. Previous studies have used closed-loop acoustic stimulation to demonstrate enhancement of neocortical slow oscillations and sleep spindles that improves memory^40,71,72^, although some studies could not replicate memory effects despite strong effects on slow waves and spindles^73,74^. One unresolved issue in all these studies is what impact the stimulation had on hippocampal ripples. In our data, we find that even though stimulation did not increase the number of ripples, the temporal co-occurrence of neocortical slow waves and hippocampal ripples is a critical predictor of memory accuracy. Possibly, the degree to which previous manipulations modulated this co-occurrence may account for the discrepancies observed in the behavioral results but, unfortunately, hippocampal ripples were not measured in those studies due to the absence of deep recording electrodes. Thus, additional studies will be required to further address the degree to which modulation of hippocampal ripples per se is necessary to induce memory benefits via stimulation.

Several features of our experimental design make it unique among previous reports. These include: (i) using intracranial electrical stimulation in frontal lobe white matter aiming to influence prefrontal–hippocampal pathways^34^; (ii) timing the stimulations in real time based on MTL slow-wave active periods, which are not necessarily in sync with slow-wave up-states as recorded on the scalp^32^; this was made possible here because of our access to deep brain iEEG signals; (iii) we developed a brief, high-frequency stimulation scheme (Methods); (iv) our setup allowed simultaneous iEEG recordings from deep brain structures to evaluate coupling between MTL ripples to slow wave and spindle events; and (v) our ability to assess the effects of intervention on neuronal spiking activities. Our results suggest that timing the interventions to moments of MTL active periods is key to achieving the memory-enhancing effects given the difference between sync stimulation and mixed-phase control stimulation. This result is in agreement both with rodent studies, pointing to cross-brain synchrony during slow-wave active states as an important factor supporting successful learning and memory consolidation^20,21,24^ and with studies in humans, supporting the idea that these states offer a privileged window for interventions targeting memory consolidation^36,40,75^. However, we do not claim that the precise timing we have chosen in this report is necessarily optimal. Additional studies should test whether synchrony could be further enhanced by refinement of the stimulation timing.

To conclude, using a rare opportunity to perform an active intracranial intervention during natural sleep and while recording detailed iEEG signals and single-unit spiking from humans, we found a tight correlation between electrophysiological signatures of NREM sleep and overnight memory consolidation. Our results support present models of systems-level consolidation, whereby functional coupling between hippocampal ripples and thalamocortical sleep spindles and slow waves mediates fine-tuned communication between the human hippocampus and neocortex during sleep. Finally, the present study suggests an approach to benefit memory consolidation during the privileged period of sleep, that may be beneficial in future development of closed-loop clinical DBS devices for memory disorders and dementia^37^.

## Methods

### Participants

Eighteen participants with pharmacologically intractable epilepsy (11 women, 7 men, based on self-reported gender identity; Supplementary Table 1) who met clinical criteria for depth electrode placement at UCLA for identification of seizure foci and preparation for surgical treatment^76^. Participants were not compensated for participation in the study. The UCLA Institutional Review Board approved the study protocol. All participants provided written consent to participate in the study. Electrode location was based solely on clinical criteria. Predetermined clinical criteria guided placement of 9–14 Behnke–Fried electrodes (Adtech Medical, Racine WI) in each individual. Electrodes were implanted stereotactically with the aid of digital subtraction angiography or computed tomography (CT) angiography as well as magnetic resonance imaging (MRI)^76^. Each Behnke–Fried macro–micro depth electrode contained at least seven macroelectrode contacts (1.5 mm wide) spaced 1.5–3.5 mm apart along the shaft, and a Behnke–Fried inner platinum-iridium microwire bundle (California Fine Wire)^76^ (Extended Data Fig. 6a(i)). All surgeries were performed by I.F. Sixteen participants were tested in two experimental sessions, as detailed below (Supplementary Table 2), while two participants underwent mixed-phase stimulation on intervention nights (without a night of undisturbed sleep), designed to serve as a control for physiological effects. Each participant’s drug regimen at the time of recording is listed in Supplementary Table 2.

### Experimental design

Participants were tested in two experimental sessions: an intervention condition (sleep with RTCL stimulation) and a control condition (undisturbed sleep), with the order of conditions and test versions (image pairs), counterbalanced across participants (Fig. 1a and Supplementary Table 2). The participants’ two experimental conditions were separated by an interval of 1–5 d (Supplementary Table 2). On the day of each experimental session, participants did not take any naps nor drank coffee in the 6 h preceding the experiment. In each condition, participants (i) first performed a declarative memory task (image-pair associates, below) between 20:00 and 22:30 (learning phase), (ii) were tested on their memory (after a short break) following learning, (iii) went to sleep; in stimulation nights, RTCL stimulation started after at least 30 min of consolidated sleep when online polysomnography indicated unequivocal NREM sleep and was discontinued 90–140 min later, and (iv) completed a memory recall examination session (retrieval phase) in the morning 30–60 min after awakening (Supplementary Table 2). Of 16 participants tested on two experimental nights, three exhibited poor memory accuracy scores (recognition memory accuracy < 0.1 in one of the tests, suggesting low attention or misunderstanding of the task, marked with an asterisk in Supplementary Table 2) and were excluded from further memory accuracy analysis (but included in neurophysiological analysis). One additional participant underwent cognitive testing but had a very low number of stimulations delivered (<100) and was excluded altogether from the cognitive cohort (Supplementary Table 2).

### Cognitive testing and overnight memory paradigm

Paired associative learning (PAL) has previously shown to be sensitive to the effect of sleep^1,40,77–79^. We adapted the task for participants to make it relatively short (20 min for the learning phase, 5–10 min for the retrieval phase), and based on visual stimuli (vPAL; see also ref. ^55^). Twenty-five color images of famous people were paired with 25 different animals (their ‘pets’). Image pairs were presented on a laptop computer at the participant’s bedside. Participants studied each image pair for 2 s followed by 2 s of fixation and were asked to memorize the pair and to name the animal’s type. After viewing each pair once, participants were given a short break in which the rest of the experiment was explained. Next, single images of people were presented for 2 s (25 learned images were mixed with 15 novel (‘lure’) images of famous people). First, participants were asked whether the person was a previously learned pet owner, without any feedback on their answer. Next, if they had identified a person as a ‘pet owner’, they were asked what kind of animal they owned (pairing). For each image, we determined whether it was correctly identified as new (lure) or old, the reaction time of that response, and whether the associated pet was correctly identified (when applicable). After an overnight sleep period (Supplementary Table 2; average delay ± standard deviation between the first and second tests, 10.7 ± 1.3 h on intervention night and 10.3 h ± 2.7 h on undisturbed nights, respectively), participants were retested (same 40 images as in the evening test, in a scrambled order) without feedback, and behavioral measures were compared for pre-sleep and post-sleep tests.

Recognition accuracy for each test was defined as:

$$\mathrm{Accuracy} = \frac{{N_{\mathrm{correct}}\;{\mathrm{recognition}}}}{{25}} - \frac{{N_{\mathrm{false}}\;{\mathrm{recognition}}}}{{15}}$$

Overnight memory change was quantified as:

$$\Delta {\mathrm{Accuracy}} = {\mathrm{Accuracy}_{morning}} - {\mathrm{Accuracy}_{evening}}$$

The efficacy of RTCL stimulation was evaluated by comparing overnight performance changes:

$$\begin{array}{l}\mathrm{Intervention}\;\mathrm{efficacy}\\ = {{\Delta }}\mathrm{Accuracy}_{\mathrm{RTCL}\;\mathrm{intervention}\;\mathrm{night}} - {{\Delta }}\mathrm{Accuracy}_{\mathrm{undisturbed}\;\mathrm{sleep}\;\mathrm{night}}\end{array}$$

This within-participant comparison was performed to distill the effects of the intervention (sleep with RTCL stimulation compared to undisturbed sleep), independent of the expected inter-participant variability in baseline long-term memory performance^31^. If the intervention had no consistent effect, we would expect by chance that the intervention night would show superior performance in about half the participants. To test this, we used the binomial cumulative distribution function (binocdf, MATLAB, MathWorks) to assess the probability of our data (observing superior performance on intervention night relative to undisturbed night for 6/6 participants), against the null hypothesis that the probability for each participant to have superior performance on stimulation nights is 0.5. To obtain estimates and 95% confidence intervals of the percentage of participants who had superior performance on intervention nights, we computed the likelihood of the data given a binomial model (binofit, MATLAB, MathWorks). To estimate confidence intervals for individual participants’ intervention efficacy, we ran a bootstrapping procedure as follows: for every test set, we selected 25 images and 15 lures (with repetitions) out of each test’s image set and calculated participant scores based on the selected set. We repeated this 1,000 times for each participant. We calculated the mean and standard deviation of the bootstrapped scores for correct recognitions, false alarms and recognition memory accuracy for each participant (plotted in Extended Data Fig. 4c–e). We also computed the mean across participants for each bootstrap iteration to estimate a distribution of means (shown in the insets of Extended Data Fig. 4c–e).

Before the evening learning session and following the morning testing session, participants performed a face/non-face categorization PVT as described in ref. ^38^ to quantify vigilance. In brief, during each block, four face images and two non-face images (places or animals) were presented on a laptop computer for 200 ms while participants performed a face/non-face categorization task. Each picture was presented 24 times in a pseudorandom order (total of 144 trials), with long pseudorandomized inter-stimulus intervals of 2–8 s (uniform distribution), as in classical PVT designs^80^. Participants were instructed to press one of two buttons (for face versus non-face) as quickly as possible. We used the difference between median reaction times between morning and evening in the visual PVT task to assess changes in vigilance following intervention nights and undisturbed nights (Extended Data Fig. 4f,g). Subjective sleepiness was assessed at the beginning of each experimental session using the Stanford Sleepiness Scale and a visual analog rating of sleepiness.

### Electrode localization

Depth electrode placement was determined solely based on clinical considerations. Before electrode implantation, we obtained for each participant a T1-weighted 1-mm isometric structural MR scan using a 3-Tesla scanner. After implantation, a CT scan was acquired and co-registered via an affine transform to the preoperative anatomical MR scan (after skull stripping) using FSL’s BET and FLIRT toolbox (FMRIB v6.0)^81–83^. This allowed visualization of the CT scan superimposed with the preoperative MRI scan (Fig. 1c and Extended Data Fig. 2). Individual recording sites were then identified visually on the co-registered CT and manually marked in each participant’s preoperative MRI native space using BioImage Suite (RRID: SCR_016109)^84^. Co-registration and electrode localization were performed using the iELVIS toolbox^84^: The preimplantation three-dimensional T1 MR scan was processed using FreeSurfer to segment the white matter, deep gray matter structures and cortex; and to parcellate the neocortex according to gyral anatomy^85,86^. Each iEEG electrode was then attributed to a cortical region according to automated parcellation in FreeSurfer^85^. We warped the aligned electrodes onto a standard brain template (using MNI template) to facilitate group-level visualization (Figs. 1d, 2d, 3c and 4b). The MNI reconstruction was performed for visualization purposes only, and electrode localizations were always determined in native MR space. All stimulation sites were verified to reside in white matter and all MTL probe locations in gray matter by a neurologist and a neurosurgeon. iEEG electrode contacts used for ripple detection (see below) were verified to be in hippocampus/entorhinal cortex/parahippocampal gyrus gray matter. We defined MTL–neocortical iEEG couples in the following manner (Fig. 4b): the ‘ripple channel’ was an MTL iEEG electrode with ripple detections in pre-stimulation sleep, while the ‘prefrontal channel’ was a prefrontal iEEG electrode with the highest number of spindle event detections during pre-stimulation sleep in the same hemisphere (in one pair/participant, we paired MTL and prefrontal contacts from opposite hemispheres). When using the MNI brain template to demonstrate widespread effects (Fig. 2d), score values outside the 5–95 percentiles are displayed in minimum/maximum colors for better visualization.

### Stimulation

A board-certified neurologist was present in each stimulation session to monitor the clinical iEEG recordings for after-discharges and ensure participant safety. Stimulation of epileptogenic areas was avoided when possible and the neurologist validated stimulation site and impedance before each session. Before every experimental session, each participant was given a short series of test stimulation pulses while a neurologist monitored the clinical iEEG recordings for after-discharges and ensuring stimulations were correctly delivered. Unaware of the exact timing of stimulation onset, participants were asked at the end of each session (in the morning) to report any unusual feelings or sensations. Participants did not report any effects of stimulation, nor could they indicate when stimulation occurred during the night. In 12/19 sessions, experiments occurred >10 h since last seizure, and in 7 sessions, experiments occurred >2 h since last seizure. A board-certified neurologist validated that no seizures were detected during intervention nights. Stimulation was current regulated and charge balanced, with pulses set below the threshold for after discharge, which was identified based on pretesting (range: 1.0–2.0 mA). Stimulation electrode impedance was measured immediately before testing (range, 1–4 kΩ, using clinical Neurofax EEG-1200A system, Nihon Koden). Stimulations were delivered in one of two schemes (Supplementary Table 3): (i) bipolar stimulation (participants 1–3) used a CereStim R96 Macro-stimulator (BlackRock Microsystems) to deliver electrical stimulation to the Behnke–Fried depth electrode bipolar macro-contacts spaced 3.5 mm apart (surface area, 0.06 cm^2^)^69^, and (ii) unipolar stimulation (participants 4–18) with the electrodes referenced to the EEG GND electrode. Each 50-ms-long stimulation event included five rectangular pulses (pulse width of 100 μs) at a frequency of 100 Hz, with the current ranging from 0.5 to 1.5 mA. Stimulation ranged between 2.5 and 7.6 μC of charge per square centimeter per phase, which is well below the safe maximum used for long-term and short-term stimulation (30 and 57 μC, respectively)^87,88^.

### Timing of real-time closed-loop stimulation by online detection of slow waves in medial temporal lobe probe

A neural signal processor (NSP; Cerebrus system, BlackRock Microsystems) connected to a separate laptop was used to detect slow waves online to time electrical stimulation events. With this setup, the predefined probe’s iEEG signal was fed to a custom code running on the laptop (MATLAB, MathWorks) in parallel to its recording by the NSP. The signal was low-pass filtered at <500 Hz and sampled at 2,000 Hz by the NSP. A custom-made script (running on MATLAB, MathWorks) using the NSP’s application programming interface (BlackRock Microsystems) enabled responding to the incoming iEEG data in real time. To this end, the iEEG signal was further band-passed filtered between 0.5 Hz and 4 Hz (50th order FIR filter). Thus, during 5-min stimulation blocks, each time the low-passed signal crossed an adaptive threshold toward larger positive values, the electrical stimulation (details above) was triggered with a delay (details below). By default, the threshold was set to 80 µV, and the delay was either based on pre-recorded sleep iEEG from participants or set to be 400 ms. The threshold was updated every 400 s to be the median iEEG amplitude of slow waves detected within the preceding a 400-s interval, and the delay from detected peak to putative active phase was updated based on average values of peak to trough of detected slow events in the same interval. This algorithm ensured a reliable way to continuously detect slow waves with dynamic amplitudes (for example, modulated by sleep depth and other factors) by their positive half-wave peaks^40^. The detection routine was resumed 3 s after the delivery of stimulation to ensure sufficient buffers to evaluate slow-wave activity between stimulation events. After recording 7–15 min of uninterrupted NREM sleep, stimulation blocks were administered in 5-min blocks, interleaved with 5-min pause blocks (‘pause’ intervals; Fig. 1b). At least 15–20 min of post-stimulation uninterrupted sleep data were recorded after the last stimulation block. In a post hoc analysis, we measured the delay between each stimulation to the MTL probe’s peak that immediately preceded it (corresponding to the down/inactive phase in iEEG signals^32^) and found the mean delay to be 241.3 ms for the sync-stimulation group and 373.3 ms for the mixed-stimulation group. We used post hoc analysis to quantify the degree to which stimulation timings were in phase with MTL active periods: We required >55% of stimulations to be within 80–280 ms of the closest peak to be included in the sync-stimulation group (Extended Data Fig. 3b). For participant 17 where post hoc analysis was not possible due to noise issues, we used the median delay from the peak based on the real-time log.

### Sleep scoring

Our approach focused on detecting epochs of NREM sleep based on iEEG signals. We were able to reliably detect NREM epochs in every recording, even when full polysomnography (which would allow reliable separation of wakefulness from REM sleep) was not available. To guide the initiation of stimulation blocks online, visual detection of NREM sleep epochs was performed at the participant bedside by a physician board certified in clinical neurophysiology. Visual detection focused on unequivocal presence of slow waves and sleep spindles in iEEG data. To guide offline detailed analysis, automatic detection of NREM sleep was performed focusing on the presence of robust slow waves and sleep spindle activities in iEEG data^89^ as follows. First, for each participant separately, we selected a neocortical iEEG channel distant from the seizure onset zone, where interictal activity was minimal, and—whenever possible—from a contact placed in medial prefrontal/parietal cortex with prominent sleep spindle occurrence^35^. Second, after removing residual interictal discharge activities (see ‘iEEG preprocessing and detection of pathological events’), we calculated the short-time Fourier transform (30-s window, no overlap, 0–40 Hz range, 0.2-Hz resolution; Fig. 1b). Note that an additional normalization by a two-dimensional Gaussian filter (σ = 3) was used for visualization purposes in the figure but not for scoring. Third, we averaged the power in the slow-wave (0.5–4 Hz) and spindle (9–16 Hz) frequency bands for each 30-s time point, resulting in two vectors representing slow-wave and spindle power. Fourth, we fit a two-component Gaussian mixture distribution to the slow-wave–spindle joint vectors to represent synchronized NREM epochs versus desynchronized (REM/wakefulness) epochs. Fifth, we calculated the posterior probability for each component given each time point (fitgmdist and posterior, MATLAB, MathWorks) and tagged each time point according to the maximal posterior probability (NREM versus desynchronized; Extended Data Fig. 1a). Finally, standalone NREM detections (30 s only) that were >1 min away from other NREM detections were discarded according to AASM guidelines^90^.

We validated our approach by comparing the automated iEEG-based scheme used here with gold-standard polysomnography-based sleep scoring performed by an expert using AASM guidelines with three pre-scored overnight recordings from an independent dataset^32^. The output of the data-driven Gaussian mixture approach was highly concordant with manual scoring (comparing the first 3 h of sleep in the three different overnight datasets, we found a <4% change in the lengths of NREM sleep bouts). The approach used here was more robust to inter-participant variability compared with other automated versions based only on predefined thresholds of delta power^89^. Importantly, post hoc analysis based on the iEEG-based sleep scoring confirmed that 76% ± 5.1% (average and s.e.m. over *n* = 19 sessions) of stimulation events occurred during NREM sleep.

### Electrophysiology data acquisition and offline spike sorting

In each participant, 8–14 depth electrodes were implanted targeting medial brain areas. Each depth electrode had eight platinum iEEG contacts along the shaft (Extended Data Fig. 6a(i), referenced to the scalp). Both scalp and depth iEEG data were continuously recorded at a sampling rate of 2 kHz, band-pass filtered between 0.1 and 500 Hz, using either Blackrock or Neuralynx data acquisition systems. Each electrode terminated in eight 40-mm platinum-iridium microwires from which extracellular signals were continuously recorded (Extended Data Fig. 6a; referenced locally to a ninth non-insulated microwire) at a sampling rate of 28 or 30 kHz and band-pass-filtered between 1 and 6,000 Hz.

#### Spike sorting

Neuronal clusters were identified using the ‘Wave Clus v2’ software package^91^ as described previously^32^: Action potentials were detected by high-pass filtering the extracellular recordings above 300 Hz and applying a threshold at 5 s.d. above the median noise level. Detected events were clustered (or categorized as noise) using automatic superparamagnetic clustering of wavelet coefficients, followed by manual refinement based on the consistency of spike waveforms and inter-spike interval distributions (see example in Extended Data Fig. 6a(iii)). Unit stability throughout stimulation sessions was confirmed by verifying that spike waveforms and inter-spike interval distributions were consistent and distinct in the interval ranging from pre-stimulation through post-stimulation time points (1–2 h; Supplementary Table 2 and Extended Data Fig. 6a(iv)). Of 386 neural clusters identified by ‘Wave Clus’ (8 patients), 325 clusters (84%) were verified to be stable throughout the session and were included in further analysis.

### Intracranial electroencephalography preprocessing and detection of pathological events

Data analysis was performed with MATLAB (MathWorks), using the FieldTrip^92^ and CircStat^93^ toolboxes as well as custom scripts. Preprocessing of the iEEG data began with line noise removal (2-Hz band-stop filters centered at 60 Hz and its harmonics) and followed by an automated algorithm to identify pathological events and electrical artifacts, as follows. First, for all data intervals occurring during NREM sleep, each time point was converted into a *z*-score based on the participant-specific and stage-specific mean and s.d. of absolute amplitude, gradient (the amplitude difference between two adjacent time points) and amplitude of the data after applying a 250-Hz high-pass filter. Next, epileptiform interictal spikes were detected automatically in iEEG signals by identifying events whose envelope of the high-passed signal was larger than a threshold of +5 s.d., or a conjunction of absolute amplitude and gradient both passing a threshold of +5 s.d. and whose duration was <70 ms^35^. Points that passed the detection condition and occurred in close temporal proximity (<50 ms) were merged as one interictal spike. Subsequent analysis of iEEG data was performed after detecting pathological events in each channel separately: we used a semi-manual process, identifying channels with gross deviations of kurtosis/amplitude/skewness relative to other channels on the same electrode and used visual validation as well as independent clinical neurologist channel-tagging to remove channels with high rates of interictal activity (>5 events per minute) or with electrical noise. In channels included in further analysis, 500 ms preceding and following any interictal spike detection were removed.

### Single-event detection of sleep oscillations

Slow waves, sleep spindle and MTL ripple events were identified independently for each participant and channel, based on established detection algorithms^35,44^.

#### Slow waves

Slow waves were detected as in ref. ^44^. First, artifact-free iEEG signals from the NREM sleep stage were filtered between 0.16 Hz and 1.25 Hz (two-pass FIR band-pass filter, order = three cycles of the low-frequency cutoff). Second, all zero-crossings were determined in the filtered signal, and event duration was determined for slow-wave candidates (that is, events consisting of an inactive/‘OFF’ period corresponding to iEEG peak, followed by an active/’ON’ period corresponding to iEEG trough^32^) as the time between two successive negative-to-positive zero-crossings. For events whose duration was between 0.8 s and 2 s, event amplitudes were determined (peak-to-trough amplitude between two negative-to-positive zero-crossings). Events that also met the amplitude criteria (≥75% percentile of candidate amplitudes, that is, the 25% of events with the largest amplitudes) were considered as slow waves.

#### Sleep spindles

Spindles were detected automatically via a two-step process based on ref. ^35^. First, to minimize false detections, only channels with robust spindle activity in NREM sleep were chosen for further analysis. To this end, in each individual channel, sigma (9–16 Hz) power in NREM sleep was compared with a fitted 1/f^α^ model (both were estimated across multiple 10-s epochs) and channels with a difference that was statistically significant at *P* < 0.001 (unpaired *t*-test for maximal peak) were further considered. Second, putative spindle events were selected based on their power and duration: iEEG signals were band-pass filtered between 9 Hz and 16 Hz using a zero-phase fourth-order Butterworth filter. The instantaneous amplitude was computed via the Hilbert transform and two thresholds were defined based on this amplitude time course across artifact-free sleep epochs. A detection threshold was set at the mean + 3 s.d. and amplitudes exceeding this threshold were considered potential spindles. A start/end threshold was set at the mean + 1 s.d., and events whose duration was between 0.5 s and 2 s were further considered. Detections within 1 s were merged as single events. We verified the spectral specificity of each spindle by excluding any detection that coincided with control events that were above the mean + 5 s.d in the 20–30 Hz range. For single-event co-occurrence analysis (see below), we used a subpopulation of fast spindles where detection required a minimum frequency > 11 Hz. For every detected spindle, the peaks and troughs were detected as the maxima and minima of the filtered signal, and the maximal peak was designated as the time point that represented the respective spindle in time (for example, for single-event co-occurrence analysis and population average).

#### Medial temporal lobe ripples

We utilized bipolar referencing to minimize effects of volume conduction by identifying, in each electrode shaft targeting the MTL separately, a contact residing in white matter to be used as a reference for single-ripple detection, using preoperative and postoperative CT and MRI data. We then used an automated detection algorithm as in ref. ^44^. First, data were filtered between 80 Hz and 100 Hz (two-pass FIR band-pass filter, order = three cycles of the low-frequency cutoff), and only artifact-free data from NREM sleep were used for event detection. Second, the root-mean-square (RMS) signal was calculated for the filtered signal using a moving average of 20 ms, and the ripple amplitude criterion was defined as the 99% percentile of RMS values. Third, whenever the signal exceeded this threshold for a minimum of 38 ms (encompassing ~3 cycles at 80 Hz) a putative ripple event was marked. In addition, to avoid sharp broadband events, only those putative ripple events representing a true oscillatory pattern were considered for further analysis. Accordingly, we focused on events with at least three discrete peaks or three discrete troughs in the raw signal corresponding to the above-threshold RMS segment. This was accomplished by identifying local maxima or minima in the respective raw signal segments after applying a one-pass moving average filter including the two adjacent data points. We demanded a detection of 20 ripples in pre-sleep baseline to include a contact as a ripple channel. Of 45 candidate MTL channels (18 participants), 17 were excluded because ripple rate was too low (13) or baseline noise was too high (7).

#### Single-event co-occurrence

Slow-wave–spindle sequences (Extended Data Fig. 5c,f) were defined similarly to ref. ^20^ as epochs where spindle peaks occurred up to 1.5 s following iEEG slow-wave positive peak (down/OFF phase) on a specific iEEG contact. We also evaluated coupling incidence of single sleep events in cross-brain electrode pairs. In each participant, we paired one contact from MTL electrodes with one contact from frontal-cortex electrode on the same hemisphere (when possible, the superior temporal gyrus was used when the frontal electrode was not available, one participant only has a pair from opposite hemispheres), with maximal spindle activity. Of 55 candidate MTL–neocortical electrode pairs (18 participants), 13 were excluded because MTL channel was excluded from ripple analysis (see above). Of the resulting 42 couples, in one pair MTL contact and neocortical contact were in different hemispheres, hence it was not included in the Fig. 4b visualization. MTL ripple–cortical slow-wave couples corresponded to epochs where ripple peaks were 50–400 ms away from the slow-wave positive peak (down/OFF phase), including cortical contacts with >10 slow waves in the evaluated period. Co-occurrence of MTL ripple and neocortical slow-wave/spindle sequences corresponded (similar to ref. ^20^) to an MTL ripple peak preceding a slow-wave–spindle sequence by 50–400 ms.

### Stimulation-locked time–frequency analysis

Stimulation-triggered analyses were performed for stimulation time points confirmed to occur during NREM sleep following post hoc sleep scoring (‘Sleep scoring’). TFRs (Figs. 2a and 4a) were extracted by calculating a spectrogram around stimulation events (0–2.5 s) and subtracting from it the pre-stimulation (−1 to 0 s) baseline spectrogram^44^. Spectrograms were calculated using ft_specest_mtmconvol (FieldTrip toolbox^92^, MATLAB, MathWorks, frequencies 5–30 Hz, 1-Hz resolution) using a sliding Hanning-tapered window with a variable, frequency-dependent, length that comprised at least five cycles^44^. Time-locked TFRs of all stimulation events were then normalized as the percentage change from pre-event baseline and were averaged for each session (Fig. 2a).

To estimate stimulation-locked average TFR increase in spindle frequency band, above and beyond the expected based on these time periods during slow-wave active phases, we generated a set of sham-stimulation points as detailed and used those to calculate a sham-locked TFR as a baseline for comparison (Fig. 2(aii)). Slow-wave peaks were detected in ‘pause’ (stimulation-free) 5-min blocks and a random subset of them was selected (equal in number to the number of real stimulations in the same session), sham events were then selected with equal delay from peaks as the stimulations in the preceding block. Time-locked TFRs of all stimulation events and sham events were normalized as the percentage change from pre-event baseline and averaged per session for each iEEG channel (Fig. 2(aii)).

Sham points were selected offline using the same algorithm as used for online timing of stimulation events, such that they also reflected MTL OFF–ON transitions, to control for the tendency of ON periods to be associated with greater spindle activity. We also performed this analysis with an alternative selection of 1,000 random sham points during ‘pause’ sessions and observed similar results.

### Single-event probability and event-rate estimation

To assess the probability of slow waves, spindles, slow-wave–spindle couples and ripples following stimulations (Figs. 2 and 4), we detected single events (see above) on each iEEG contact separately. To evaluate the immediate effect of stimulation on sleep oscillations (Fig. 2a and Extended Data Figs. 5a–c and 9a), we counted the detections during the 3 s following stimulation events (for slow waves and spindles) and during 200 ms following stimulation events (for ripples). Probabilities were calculated as the sum of detections during immediate short periods following stimulation bursts, divided by the number of stimulations in each session vectors. Note that for 3-s time vectors, event rates and probability values were very similar for slow waves and spindles, as they typically do not occur more than once during these time periods^35^. As a within-session control, we used an equal number of sham time points (explained in the previous section, above) to assess the degree to which stimulation increased event-detection probabilities, beyond the endogenous rates during active phases of slow waves. iEEG contacts with no detections in one of the terms were excluded from the analysis. We normalized this change by contrasting the probability (P) to detect an event following stimulation with the probability calculated for sham time points: $\frac{{P_{\mathrm{stim}} - P_{\mathrm{sham}}}}{{P_{\mathrm{stim}} + P_{\mathrm{sham}}}}$. Spindle enhancement score per participant (Fig. 2d) was defined as the median of all spindle-increase scores for all iEEG contacts, excluding contacts that did not have any spindle detections in either one of the conditions (stim/sham).

For longer time epochs (the prolonged condition in Figs. 2 and 4 and Extended Data Figs. 5, 8 and 9), event rates were calculated as the number of detected events divided by the accumulated length of time. Event enhancement score in the prolonged condition (Fig. 2d) was similarly calculated as a contrast index between post-stimulation time vectors (1 min post-stimulation block) and the furthest equal-length time period in the remaining ‘pause’ block (that is, 1 min before the following stimulation block): $\frac{\mathrm{Event}\;\mathrm{rate}_{\mathrm{post} - \mathrm{stim}} - \mathrm{Event}\;\mathrm{rate}_{\mathrm{pre} - \mathrm{stim}}}{{\mathrm{Event}\;\mathrm{rate}_{\mathrm{post} - \mathrm{stim}} + \mathrm{Event}\;\mathrm{rate}_{\mathrm{pre} - \mathrm{stim}}}}$.

### Estimating phase locking of neural spiking activity by fitting a cosine function

All analyses were performed using MATLAB (MathWorks). To quantify the degree of phase locking between MTL slow-wave oscillations and neuronal spiking activity in different brain regions (Fig. 3), we fitted a cosine function to the distribution of spike phases relative to phase values of the MTL probe’s iEEG signal^94^. For each neural cluster and each condition (baseline, stimulation as described below), we repeated the fitting procedure to create a unique lock-depth measure for each condition (Extended Data Fig. 6b). First, we computed the instantaneous amplitude of the MTL probe’s iEEG signal via the Hilbert transform following a Butterworth band-pass filter between 0.5 Hz and 2 Hz (zero-phase filtering via filtfilt). We then extracted the probe’s phase for each neural spike. We used 20-degree bins to create a histogram of spike phases (−180 < *φ* < 180). We fitted every spike-phase histogram with the follomatlabwing function: $f(\varphi ) = a \times \cos (\varphi + b) + c$ (Extended Data Fig. 6b). We computed the *R*^2^ value between the original spike-histogram and the fit. We found *R*^2^ > 0.25 to have a good correspondence with the Rayleigh test for non-uniformity of phases (calculated by circ_rtest), as >90% of distributions over this value had passed *P* < 0.05, but the fitting procedure was less sensitive to variations in distribution shape than the circular statistics.

We defined an index to capture the phase-locking depth (LD) of oscillatory modulation in our fit as follows: $\mathrm{LD} = \frac{{2a}}{{(2 \times a + c)}}$.

We included in our analysis 325 neural units validated as having a stable inter-spike-interval probability distribution (see above). For each condition, we analyzed phase locking for neural units with a minimum mean firing rate of 0.1 Hz. We included in the phase-locking change analysis (Fig. 3c and Extended Data Fig. 6c) neural units with significant phase locking during evaluated conditions (Rayleigh test, *P* < 0.05). To describe in full the immediate and prolonged changes in phase locking, we defined several conditions for the evaluation of spiking activity. To assess the change during stimulation blocks, baseline values were based on neural activity during the uninterrupted 5-min sleep period before the first intervention (‘PRE’; Fig. 1b). During stimulation blocks (used in Fig. 3b), we excluded spikes that occurred within the 500 ms following stimulation events. We defined a prolonged condition as all 1-min post-stimulation blocks. Phase-locking depth change was evaluated by the following index (Fig. 3c,d and Extended Data Fig. 6c, d): $\frac{{\mathrm{LD}_{\mathrm{post} - \mathrm{stim}} - \mathrm{LD}_{\mathrm{baseline}}}}{{\mathrm{LD}_{\mathrm{post} - \mathrm{stim}} + \mathrm{LD}_{\mathrm{baseline}}}}$.

We compared locking depth for units that had significant phase locking in compared conditions (Rayleigh test, *P* < 0.05). Note that the number of clusters with significant phase locking increased during the stimulation session, as demonstrated in Fig. 3b. These changes resulted in a varying number of units for each evaluated condition. We calculated lock-depth change for all various combinations of evaluated conditions versus the two different baselines used in the main text (‘PRE’ sleep and 1-min pre-stimulation blocks), all in agreement with the main analysis reported in Fig. 3c,d (see full distributions and statistics in Extended Data Fig. 6c).

We performed two separate shuffling procedures to validate the prolonged effect in phase locking—comparing the prolonged condition to the 1-min period before stimulation blocks (Fig. 3d): (1) We assigned lock-depth values to the tested condition or baseline randomly (randperm, MATLAB, MathWorks) 10,000 times and tested whether the randomly shuffled distribution differed significantly from the calculated distribution (Extended Data Fig. 6c). (2) To test against the hypothesis that changes in firing rates bias our phase-locking calculation, we performed the following procedure 1,000 times—for each comparison between conditions, we selected a set of *X* spikes from each condition; *X* totaled 90% of the minimum spike count of the two conditions. We refit a cosine as described above for both selected sets and recalculated phase-locking change. The resulting distribution was not significantly different from the distribution calculated based on the full number of spikes and significantly passed the Wilcoxon sign-rank test as the distribution reported in Fig. 3d (Extended Data Fig. 6c).

### Statistical analyses

We used parametric methods for statistical testing of normal data. For non-normal data or small sample sizes, we used Wilcoxon signed-rank/rank-sum tests. To compare two distributions, we used the Kolmogorov–Smirnov two-sample test. All statistical tests were two sided unless stated otherwise. In violin plots representing estimated distributions of data (generated with violinplot, FieldTrip toolbox^92^, MATLAB, MathWorks), lines represent 5, 50 and 95 percentiles. No statistical methods were used to predetermine sample sizes but our sample sizes are similar to those generally used in previous publications^44,73,95^. Data collection and analysis were not performed blind to the conditions of the experiments.

### Reporting summary

Further information on research design is available in the Nature Portfolio Reporting Summary linked to this article.

## Online content

Any methods, additional references, Nature Portfolio reporting summaries, source data, extended data, supplementary information, acknowledgements, peer review information; details of author contributions and competing interests; and statements of data and code availability are available at 10.1038/s41593-023-01324-5.

## Supplementary information

Supplementary Information Supplementary Tables 1–4

Reporting Summary

## Data Availability

Supplementary tables supporting the findings of this paper are available as Supplementary Information. Source data are provided with this paper.

## Source data

Source Data Fig. 1 Statistical source data.
Source Data Fig. 2 Statistical source data.
Source Data Fig. 3 Statistical source data.
Source Data Fig. 4 Statistical source data.
Source Data Extended Data Fig. 1 Statistical source data.
Source Data Extended Data Fig. 3 Statistical source data.
Source Data Extended Data Fig. 4 Statistical source data.
Source Data Extended Data Fig. 5 Statistical source data.
Source Data Extended Data Fig. 6 Statistical source data.
Source Data Extended Data Fig. 7 Statistical source data.
Source Data Extended Data Fig. 8 Statistical source data.
Source Data Extended Data Fig. 9 Statistical source data.
Source Data Extended Data Fig. 10 Statistical source data.

## Code availability

Toolboxes developed by authors for the detection of single events in iEEG data are freely available on GitHub:

https://github.com/mgevasagiv/sleepScoringIEEG, sleep scoring based on iEEG data;

https://github.com/mgevasagiv/rippleDetection_IEEG, ripple detection in iEEG data;

https://github.com/mgevasagiv/epilepticActivity_IEEG, IED detection in iEEG data;

https://github.com/mgevasagiv/sleepOscillations_IEEG, slow-wave and spindle detection in iEEG data.

Additional code used for data analysis is available on reasonable request from the corresponding authors.

## References

[1] Jenkins JG, Dallenbach KM (1924). Obliviscence during sleep and waking. Am. J. Psychol..

[2] Maquet P (2001). The role of sleep in learning and memory. Science.

[3] Walker MP, Stickgold R (2004). Sleep-dependent learning and memory consolidation. Neuron.

[4] Diekelmann S, Born J (2010). The memory function of sleep. Nat. Rev. Neurosci..

[5] Squire LR, Zola SM (1996). Structure and function of declarative and nondeclarative memory systems. Proc. Natl Acad. Sci. USA.

[6] Marr D (1971). Simple memory: a theory for archicortex. Philos. Trans. R. Soc. L. B Biol. Sci..

[7] Buzsáki G (1989). Two-stage model of memory trace formation: a role for ‘noisy’ brain states. Neuroscience.

[8] Buzsáki G (1996). The hippocampo–neocortical dialogue. Cereb. Cortex.

[9] Vaz AP, Inati SK, Brunel N, Zaghloul KA (2019). Coupled ripple oscillations between the medial temporal lobe and neocortex retrieve human memory. Science.

[10] Liu AA (2022). A consensus statement on detection of hippocampal sharp wave ripples and differentiation from other fast oscillations. Nat. Commun..

[11] Girardeau G, Lopes-Dos-Santos V (2021). Brain neural patterns and the memory function of sleep. Science.

[12] Logothetis NK (2012). Hippocampal–cortical interaction during periods of subcortical silence. Nature.

[13] Skelin I (2021). Coupling between slow waves and sharp-wave ripples engages distributed neural activity during sleep in humans. Proc. Natl Acad. Sci. USA.

[14] Steriade M (2006). Grouping of brain rhythms in corticothalamic systems. Neuroscience.

[15] Timofeev I (2011). Neuronal plasticity and thalamocortical sleep and waking oscillations. Prog. Brain Res..

[16] Klinzing JG, Niethard N, Born J (2019). Mechanisms of systems memory consolidation during sleep. Nat. Neurosci..

[17] Ohki T, Takei Y (2018). Neural mechanisms of mental schema: a triplet of delta, low beta/spindle and ripple oscillations. Eur. J. Neurosci..

[18] Lewis PA, Cairney S, Manning L, Critchley HD (2011). The impact of overnight consolidation upon memory for emotional and neutral encoding contexts. Neuropsychologia.

[19] Talamini LM, Nieuwenhuis ILC, Takashima A, Jensen O (2008). Sleep directly following learning benefits consolidation of spatial associative memory. Learn. Mem..

[20] Maingret N, Girardeau G, Todorova R, Goutierre M, Zugaro M (2016). Hippocampo–cortical coupling mediates memory consolidation during sleep. Nat. Neurosci..

[21] Latchoumane CV, Ngo HV, Born J, Shin HS (2017). Thalamic spindles promote memory formation during sleep through triple-phase locking of cortical, thalamic, and hippocampal rhythms. Neuron.

[22] Siapas AG, Wilson MA (1998). Coordinated interactions between hippocampal ripples and cortical spindles during slow-wave sleep. Neuron.

[23] Binder S (2019). Monosynaptic hippocampal–prefrontal projections contribute to spatial memory consolidation in mice. J. Neurosci..

[24] Benthem SD (2020). Impaired hippocampal–cortical interactions during sleep in a mouse model of Alzheimer’s disease. Curr. Biol..

[25] Girardeau G, Benchenane K, Wiener SI, Buzsáki G, Zugaro MB (2009). Selective suppression of hippocampal ripples impairs spatial memory. Nat. Neurosci..

[26] Ego‐Stengel V, Wilson MA (2010). Disruption of ripple‐associated hippocampal activity during rest impairs spatial learning in the rat. Hippocampus.

[27] Fernandez-Ruiz A (2019). Long-duration hippocampal sharp wave ripples improve memory. Science.

[28] Helfrich RF (2019). Bidirectional prefrontal–hippocampal dynamics organize information transfer during sleep in humans. Nat. Commun..

[29] Sanda P (2021). Bidirectional interaction of hippocampal ripples and cortical slow waves leads to coordinated spiking activity during NREM sleep. Cereb. Cortex.

[30] Yonelinas AP, Ranganath C, Ekstrom AD, Wiltgen BJ (2019). A contextual binding theory of episodic memory: systems consolidation reconsidered. Nat. Rev. Neurosci..

[31] Hoppe C, Elger CE, Helmstaedter C (2007). Long-term memory impairment in patients with focal epilepsy. Epilepsia.

[32] Nir Y (2011). Regional slow waves and spindles in human sleep. Neuron.

[33] Sirota A, Csicsvari J, Buhl D, Buzsáki G (2003). Communication between neocortex and hippocampus during sleep in rodents. Proc. Natl Acad. Sci. USA.

[34] Eichenbaum H (2017). Prefrontal–hippocampal interactions in episodic memory. Nat. Rev. Neurosci..

[35] Andrillon T (2011). Sleep spindles in humans: insights from intracranial EEG and unit recordings. J. Neurosci..

[36] Geva-Sagiv M, Nir Y (2019). Local sleep oscillations: implications for memory consolidation. Front. Neurosci..

[37] Mankin EA, Fried I (2020). Modulation of human memory by deep brain stimulation of the entorhinal–hippocampal circuitry. Neuron.

[38] Nir Y (2017). Selective neuronal lapses precede human cognitive lapses upon sleep deprivation. Nat. Med..

[39] Gais S, Mölle M, Helms K, Born J (2002). Learning-dependent increases in sleep spindle density. J. Neurosci..

[40] Ngo HV, Martinetz T, Born J, Mölle M (2013). Auditory closed-loop stimulation of the sleep slow oscillation enhances memory. Neuron.

[41] Bar E (2020). Local targeted memory reactivation in human sleep. Curr. Biol..

[42] Rasch B, Born J (2013). About sleep’s role in memory. Physiol. Rev..

[43] Steriade M, Nunez A, Amzica F (1993). Intracellular analysis of relations between the slow (<1 Hz) neocortical oscillation and other sleep rhythms of the electroencephalogram. J. Neurosci..

[44] Staresina BP (2015). Hierarchical nesting of slow oscillations, spindles and ripples in the human hippocampus during sleep. Nat. Neurosci..

[45] Bragin A, Engel J, Wilson CL, Fried I, Buzsáki G (1999). High-frequency oscillations in human brain. Hippocampus.

[46] Clemens Z (2007). Temporal coupling of parahippocampal ripples, sleep spindles and slow oscillations in humans. Brain.

[47] Sakon JJ, Kahana MJ (2022). Hippocampal ripples signal contextually mediated episodic recall. Proc. Natl Acad. Sci. USA.

[48] Mölle M, Bergmann TO, Marshall L, Born J (2011). Fast and slow spindles during the sleep slow oscillation: disparate coalescence and engagement in memory processing. Sleep.

[49] Schabus M (2007). Hemodynamic cerebral correlates of sleep spindles during human non-rapid eye movement sleep. Proc. Natl Acad. Sci. USA.

[50] Vyazovskiy VV, Faraguna U, Cirelli C, Tononi G (2009). Triggering slow waves during NREM sleep in the rat by intracortical electrical stimulation: effects of sleep/wake history and background activity. J. Neurophysiol..

[51] Suthana N (2012). Memory enhancement and deep brain stimulation of the entorhinal area. N. Engl. J. Med..

[52] Rajasethupathy P, Ferenczi E, Deisseroth K (2016). Targeting neural circuits. Cell.

[53] Mohan UR (2020). The effects of direct brain stimulation in humans depend on frequency, amplitude, and white-matter proximity. Brain Stimul..

[54] Mayberg HS (2005). Deep brain stimulation for treatment-resistant depression. Neuron.

[55] Schmidig, F. et al. A visual paired associate learning (vPAL) paradigm to study memory consolidation during sleep. Preprint at *bioRxiv*10.1101/2023.03.28.534494 (2023). 10.1111/jsr.14151 PMC12303256 38286437

[56] Jacoby LL, Shimizu Y, Daniels KA, Rhodes MG (2005). Modes of cognitive control in recognition and source memory: depth of retrieval. Psychon. Bull. Rev..

[57] Payne JD (2009). The role of sleep in false memory formation. Neurobiol. Learn. Mem..

[58] Diekelmann S, Born J, Wagner U (2010). Sleep enhances false memories depending on general memory performance. Behav. Brain Res..

[59] Huan S-Y, Xu H-Z, Wang R, Yu J (2022). The different roles of sleep on false memory formation between young and older adults. Psychol. Res..

[60] Nir, Y., Le Van Quyen, M., Tononi, G. & Staba, R. J. Microelectrode studies of human sleep. in *Single Neuron Studies of the Human Brain: Probing Cognition* (eds. I. Fried et al.) 65–188 (The MIT Press, 2014).

[61] Crespel A, Coubes P, Baldy-Moulinier M (2000). Sleep influence on seizures and epilepsy effects on sleep in partial frontal and temporal lobe epilepsies. Clin. Neurophysiol..

[62] Bazil CW (2003). Effects of antiepileptic drugs on sleep structure: are all drugs equal?. CNS Drugs.

[63] Boly M (2017). Altered sleep homeostasis correlates with cognitive impairment in patients with focal epilepsy. Brain.

[64] Klimes P (2019). NREM sleep is the state of vigilance that best identifies the epileptogenic zone in the interictal electroencephalogram. Epilepsia.

[65] Steriade M, Contreras D, Amzica F (1994). Synchronized sleep oscillations and their paroxysmal developments. Trends Neurosci..

[66] Gelinas JN, Khodagholy D, Thesen T, Devinsky O, Buzsáki G (2016). Interictal epileptiform discharges induce hippocampal-cortical coupling in temporal lobe epilepsy. Nat. Med..

[67] Ezzyat Y (2018). Closed-loop stimulation of temporal cortex rescues functional networks and improves memory. Nat. Commun..

[68] Titiz AS (2016). Theta-burst microstimulation in the human entorhinal area improves memory specificity. Elife.

[69] Mankin EA (2021). Stimulation of the right entorhinal white matter enhances visual memory encoding in humans. Brain Stimul..

[70] Marshall L, Born J (2007). The contribution of sleep to hippocampus-dependent memory consolidation. Trends Cogn. Sci..

[71] Ong JL (2016). Effects of phase-locked acoustic stimulation during a nap on EEG spectra and declarative memory consolidation. Sleep. Med..

[72] Leminen MM (2017). Enhanced memory consolidation via automatic sound stimulation during non-REM sleep. Sleep.

[73] Henin, S. et al. Closed-loop acoustic stimulation enhances sleep oscillations but not memory performance. *eNeuro* **6**, ENEURO.0306-19.2019 (2019). 10.1523/ENEURO.0306-19.2019 PMC6831893 31604814

[74] Cordi MJ, Rasch B (2021). How robust are sleep-mediated memory benefits?. Curr. Opin. Neurobiol..

[75] Batterink LJ, Creery JD, Paller KA (2016). Phase of spontaneous slow oscillations during sleep influences memory-related processing of auditory cues. J. Neurosci..

[76] Fried I (1999). Cerebral microdialysis combined with single-neuron and electroencephalographic recording in neurosurgical patients. J. Neurosurg..

[77] Marshall L, Mölle M, Hallschmid M, Born J (2004). Transcranial direct current stimulation during sleep improves declarative memory. J. Neurosci..

[78] Plihal W, Born J (1997). Effects of early and late nocturnal sleep on declarative and procedural memory. J. Cogn. Neurosci..

[79] Tadros T, Bazhenov M (2022). Role of sleep in formation of relational associative memory. J. Neurosci..

[80] Lim J, Dinges DF (2008). Sleep deprivation and vigilant attention. Ann. N. Y. Acad. Sci..

[81] Jenkinson M, Smith S (2001). A global optimisation method for robust affine registration of brain images. Med. Image Anal..

[82] Jenkinson M, Bannister P, Brady M, Smith S (2002). Improved optimization for the robust and accurate linear registration and motion correction of brain images. Neuroimage.

[83] Smith SM (2002). Fast robust automated brain extraction. Hum. Brain Mapp..

[84] Groppe DM (2017). iELVis: An open source MATLAB toolbox for localizing and visualizing human intracranial electrode data. J. Neurosci. Methods.

[85] Desikan RS (2006). An automated labeling system for subdividing the human cerebral cortex on MRI scans into gyral based regions of interest. Neuroimage.

[86] Fischl B (2012). FreeSurfer. Neuroimage.

[87] Gordon B (1990). Parameters for direct cortical electrical stimulation in the human: histopathologic confirmation. Electroencephalogr. Clin. Neurophysiol..

[88] Agnew WF, McCreery DB (1990). Considerations for safety with chronically implanted nerve electrodes. Epilepsia.

[89] Ramot M (2013). Emergence of sensory patterns during sleep highlights differential dynamics of REM and non-REM sleep stages. J. Neurosci..

[90] Iber, C., Ancoli-Israel, S., Chesson, A. L. & Quan, S. F. *AASM manual for the scoring of sleep and associate events. Rules, terminology and technical specifications* (American Association of Sleep Medicine, 2007).

[91] Quiroga RQ, Nadasdy Z, Ben-Shaul Y (2004). Unsupervised spike detection and sorting with wavelets and superparamagnetic clustering. Neural Comput..

[92] Oostenveld R, Fries P, Maris E, Schoffelen J-M (2011). FieldTrip: open source software for advanced analysis of MEG, EEG, and invasive electrophysiological data. Comput. Intell. Neurosci..

[93] Berens P (2009). CircStat: a MATLAB toolbox for circular statistics. J. Stat. Softw..

[94] Eliav T (2018). Nonoscillatory phase coding and synchronization in the bat hippocampal formation. Cell.

[95] Lafon B (2017). Low frequency transcranial electrical stimulation does not entrain sleep rhythms measured by human intracranial recordings. Nat. Commun..

