# Supplementary material for: Augmenting hippocampal–prefrontal neuronal synchrony during sleep enhances memory consolidation in humans
Source: Nat Neurosci. 2023 Jun 1;26(6):1100–10. doi: 10.1038/s41593-023-01324-5 (PMC10244181; doi:10.1038/s41593-023-01324-5)
Supplement: Supplementary file 1 — Supplementary Tables 1–4 [file 41593_2023_1324_MOESM1_ESM.pdf]

nature neuroscience

Article

https://doi.org/10.1038/s41593-023-01324-5

Augmenting hippocampal–prefrontal neuronal synchrony during sleep enhances memory consolidation in humans

In the format provided by the
authors and unedited

ID	Age	Gender	Handedness	Seizure Onset	FDG-PET	MRI
**1**	23	W	R	Broad right fronto-temporal onset	RPHG and R-parietal hypometabolism	Normal
**2**	33	M	R	R-frontal lobe and R preSMA	Normal	Normal
**3**	33	W	R	Left mesial temporal lobe	LTL hypometabolism	Normal
**4**	38	M	L	R mesial temporal lobe	R-temporal and R-parietal hypometabolism	Structural abnormalities right superior parietal and temporal lobe
**5**	47	W	R	LITG	LTL hypometabolism	LTL structural abnormality
6	34	W	R	Right Occipital	R basal occipital and R-PNH hypometabolism	Bilateral PNH
**7**	36	M	R	R temporo-occipital	Normal	Normal
**8**	34	W	R	Bilateral temporal	Bilateral MTL hypometabolism	Bilateral hippocampal sclerosis
**9**	29	W	R	Bilateral temporal (R>L)	R-temporal hypometabolism	Normal
**10**	44	M	R	Broad R frontal and R parietal lobes, and RINS	R-parieto-occipital hypometabolism	Normal
**11**	35	M	R	LFSG and LINS	LTL hypometabolism	Normal
**12**	31	W	R	L SMA	Normal	Normal
**13**	25	W	R	R MTL	LTL hypometabolism	L-hippocampal sclerosis
**14**	32	M	R	Bilateral MTL	LTL hypometabolism	L-hippocampal sclerosis
**15**	22	W	R	Mesial LSTG, mesial LHSG	Normal	Normal
**16**	25	W	R	Bilateral temporal lobes	Left temporal hypometabolism	Subependymal gray matter heterotopia (periventricular)
**17**	32	W	R	Left temporal lobe	Normal	L-hippocampal sclerosis
**18**	19	M	R	Left frontal (heterotopia)	Left frontal	Left frontal heterotopia

Supplementary Table 1. Patient demographics and clinical information. Columns: (i) Participant id, (ii) Age, (iii) Gender (self reported, M/W = Man/Woman), (iv) Handedness (R/L = Right/Left), (v) Seizure onset area; (vi) Imaging FDG-PET and (vii) MRI. Abbreviations: R = right hemisphere, L = Left hemisphere; AFSG – anterior fusiform gyrus, INS – insula, TL – temporal lobe, STG – superior temporal gyrus, HSG – Heschel's gyrus, MTL – medial temporal lobe, PHG –parahippocampal gyrus, PNH – periventricular nodular heterotopia, SMA – supplementary motor area.

(i) Participant id	(ii) ORDER (CL = closed loop stimulation; UD = Undisturbed night; X Days delay between sessions)	(iii) REAL-TIME CLOSED LOOP SESSION (CL)											(iv) UNDISTURBED SESSION (UD)									(v) Medications	
		1. Vigilance test (PRE)	2. Learning and baseline testing	3. Image set version	4. Sleep	5. Stimulation session start	6. Stim Duration (mins)	7. Wake up	8. Post sleep testing	9. Vigilance test (POST)	10. Time elapsed (min) - Evening learning to morning testing	11. Time Elapsed (min) - waking up to morning testing	1. Vigilance test (PRE)	2. Learning and baseline testing	3. Image set version	4. Sleep	5. Wake	6. Post sleep testing	7. Vigilance test (POST)	8. Time elapsed (min) - Evening learning to morning testing	9. Time Elapsed (min) - waking up to morning testing	1. CL night	2. UD night
**1***	UD-CL (2D)	10:15PM	10:26PM	2	11:45PM	12:15AM	90	7:43AM	8:10 AM	8:30AM	584	27	11:00 PM	11:30PM	1	2:10AM	8:10AM	8:25AM	8:35AM	536	15	Vimpat, Brivaracetam	Vimpat , Brivaracetam, Oxycodone
**2**	UD-CL (1D)	9:05PM	9:10PM	1	11:45PM	12:09AM	90	5:15 AM	6:10 AM	6:30AM	541	55	3:10PM	3:31PM	2	3:45PM	5:30PM	5:40PM	5:56PM	129	10	Vimpat, Keppra	
**3**	UD-CL (3D)	8:30PM	8:45PM	1	11:00 PM	11:30PM	120	5:50AM	6:40 AM	6:50AM	607	50	8:45PM	9:04PM	2	10:00PM	5:30 AM	6:34AM	N/A	570	65	Oxcarbazepine	Onfi, Oxcarbazepine
**4**	CL-UD (1D)	10PM	11:15PM	2	12:20AM	2:00:00AM	90	7:55AM	8:45 AM	8:55AM	570	50	9:00 PM	8:38PM	3	2:30AM	8:50AM	9:15AM	N/A	757	25	Onfi, Keppra, Lamotrigine, Benadryl	Lamotrigine, Benadryl
**5**	CL-UD (5D)	8:50PM	9:15PM	1	11:00 PM	12:05AM	90	9:00AM	9:30 AM	N/A	763	30	N/A	10:07PM	2	10:30PM	7:00AM	10:20AM	N/A	733	200		Dilantin, Oxycodone
**7**	CL-UD (3D)	8:45PM	9:10PM	1	11:00 PM	11:35PM	150	6:15AM	7:15 AM	7:25AM	605	60	8:10PM	8:30PM	3	10:00PM	6:45AM	7:20AM	7:30AM	650	35		
**8**	CL-UD (1D)	N/A	8:40PM	2	10:00 PM	10:50PM	160	8:10AM	9:05 AM	N/A	745	55	8:35PM	8:55PM	1	10:15PM	9:15AM	9:50AM	10AM	775	35	Aptiom, Brivaracetam	
**9**	CL-UD (1D)	9PM	9:20PM	2	10:00 PM	11:00 PM	120	8:30AM	8:55 AM	9:05AM	695	25	7:30PM	7:50PM	1	10PM	8:30AM	9:30AM	10:10AM	820	60	Oxcarbazepine	
**10**	UD**-CL-UD (1D)	9:30PM	9:45PM	1	12:00 AM	12:25AM	100	7:15AM	8:00 AM	8:20AM	615	45	9:05 PM	9:10PM	3	11:00 PM	6:30AM	8:30 AM	8:50AM	680	120	Vimpat, Lamotrigine, Oxycodone	Vimpat, Lamotrigine, Oxycodone
**12**	CL-UD (4D)	9:30PM	9:40PM	1	1:10AM	2:15AM	60	9:00 AM	9:50 AM	10:10 AM	730	70	9:30PM	9:45PM	3	12:30AM	8:35AM	9:50AM	10:05AM	725	75	Lamotrigine, Oxycodone	Brivaracetam, Lamotrigine, Oxycodone
**13***	UD-CL (1D)	11:30PM	11:45PM	3	1:15AM	2:35AM	60	8:30AM	9:30AM	9:45AM	585	60	11:30PM	11:40 PM	2	12:00AM	8:30AM	9:50 AM	11:07 AM	610	80	Hydroxygine	Hydromorphone, Oxycodone
**14**	CL-UD (1D)	10:25PM	10:50PM	2	12:45AM	3:45AM	45	8:00 AM	8:50AM	9:05AM	600	50	11:10PM	11:30PM	1	12:00AM	8:15AM	8:45AM	9:05AM	555	30	Clobazam, Lamotrigine, Oxycodone	Clobazam, Lamotrigine, Oxycodone
**15**	UD-CL (3D)	10:11AM	10:22PM	2	10:50PM	12:05AM	59	7:45AM	9:19AM	9:28AM	657	94	8:35PM	9:09PM	1	10:00PM	7:00AM	8:43AM	9:07AM	694	103		
**16*****	UD-CL (9D)	21:58PM	10:15PM	2	12:00AM	12:15PM	57	7:30AM	8:15AM	8:28AM	600	45	9:36PM	9:51PM	3	11:00PM	6:30AM	7:44AM	7:51 AM	607	74	Oxycodone	
**17*,*****	UD-CL (1D)	23:33PM	12:00AM	1	12:25AM	1:42AM	35	9:05AM	9:45AM	9:56AM	585	40	1:01AM	01:46AM	2	2:35AM	8:50AM	10:30AM	10:38AM	524	100	Hydromorphone, Oxycodone, methoocarbomol	Hydromorphone, Oxycodone, methoocarbomol
**18**	CL-UD (1D)	20:48PM	09:00PM	1	10:50PM	12:04AM	55	08:20AM	10:08AM	10:21AM	788	110	10:32PM	10:40PM	2	12:30AM	08:34AM	09:40AM	9:50AM	660	66	Oxycodone, fluxetine	Fluxetine

* Pts were excluded from cognitive analysis based on poor performance (Methods)
** First undisturbed-night morning follow-up not completed due to technical reasons
*** Pt excluded for low number of stimulations (Methods)

Supplementary Table 2. Cognitive testing details

Columns: (i) Participant-id of subset of participants with cognitive testing, asterisks denotes participants who were excluded from memory accuracy statistics but included in neurophysiology analysis (Methods, patients 1,13,16-17). (ii) Order of testing nights – CL = RTCL intervention night, UD = undisturbed night. In parentheses are the number of days between experimental nights. (iii) Sub-columns for each intervention experimental night – time of day (TOD) for (1) vigilance testing; (2) learning and testing in memory paradigm; (3) test version (3 different sets of images); (4) Sleep start TOD as recorded by an observer based on participant's EEG signals; (5) Stimulation session start TOD; (6) Stimulation session duration (comprised as intermittent stimulation and pause blocks as described in Fig. 1b); (7) Wake up TOD; (8) morning testing TOD; Time elapsed (min) between (9) evening learning to morning testing and (10) wake-up to testing. (iv) Sub-columns for each un-disturbed experimental night – same as (iii) without the stimulation-related columns. (v) Participant's drug regimen during (1) CL intervention nights and (2) undisturbed nights Shading corresponds to stimulation type (see Supplementary Table 4) – sync stimulation in orbitofrontal cortex (red) or other areas (brown), and mixed-phase stimulation (gray).

	UNDISTURBED NIGHT											INTERVENTION NIGHT											
	Evening Test				Morning Test							Evening Test				Morning Test							
Pt ID	imageCorrectPairEve	imageFalsePairEve	imageCorrectRecognitionEve	imageFalseDetectionEve	imageCorrectPairMor	imageFalsePairMor	imageCorrectRecognitionMor	imageFalseDetectionMor	Mem Acc evening	Mem Acc morning	diff	imageCorrectPairEve	imageFalsePairEve	imageCorrectRecognitionEve	imageFalseDetectionEve	imageCorrectPairMor	imageFalsePairMor	imageCorrectRecognitionMor	imageFalseDetectionMor	Mem Acc evening	Mem Acc morning	diff	diff of nights
2	10	4	23	0	9	7	20	0	0.92	0.80	-0.12	15	3	24	0	10	8	24	0	0.96	0.96	0.00	0.12
3	6	10	25	0	3	8	23	0	1.00	0.92	-0.08	3	12	20	1	2	15	19	1	0.73	0.69	-0.04	0.04
4	0	0	16	0	0	0	11	3	0.64	0.24	-0.40	8	3	13	0	3	4	10	0	0.52	0.40	-0.12	0.28
5	4	3	20	1	3	5	17	3	0.73	0.48	-0.25	9	8	22	1	2	4	15	4	0.81	0.33	-0.48	-0.23
7	9	0	23	0	8	0	18	1	0.92	0.65	-0.27	11	0	23	0	8	0	20	1	0.92	0.73	-0.19	0.08
8	0	13	13	1	1	13	14	2	0.45	0.43	-0.03	1	15	16	2	2	18	20	8	0.51	0.27	-0.24	-0.21
9	5	11	18	1	2	12	16	2	0.65	0.51	-0.15	1	14	15	1	2	9	11	0	0.53	0.44	-0.09	0.05
10	15	10	24	0	11	14	25	0	0.96	1.00	0.04	17	8	25	0	15	10	25	2	1.00	0.87	-0.13	-0.17
12	13	3	16	0	9	4	14	0	0.64	0.56	-0.08	12	3	23	0	6	4	17	1	0.92	0.61	-0.31	-0.23
14	12	5	20	0	8	9	17	1	0.80	0.61	-0.19	9	2	19	1	8	4	15	1	0.69	0.53	-0.16	0.03
15	11	12	24	1	10	11	24	2	0.89	0.83	-0.07	12	9	23	0	10	13	23	1	0.92	0.85	-0.07	0.00
18	12	7	20	0	9	6	19	2	0.80	0.63	-0.17	12	7	20	0	9	4	20	1	0.80	0.73	-0.07	0.11

Supplementary Table 3. Cognitive testing (Raw data). Columns: (1) Participant-id of a subset of participants included in the cognitive testing cohort (exclusion criteria detailed in Methods), (2) number of correct image-pairs recalled in evening test; Evening test: (3) number of falsely recalled image-pairs; (4) number of correctly recognized images; (5) number of falsely recognized lures in evening test; (6-9) Same assessments for morning test; (10) Recogntion memory accuracy (Methods) at evening test; (11) Recogntion memory accuracy (Methods) at morning test; (12) difference between recognition memory accuracy between morning and evening. Rightmost column is the difference between the differences measured over intervention night and undisturbed night.

Participant id	Session id	Start of session time of day	N stimulation blocks	Total session length (minutes)	Stimulation type (b = bipolar, u = unipolar)	Stimulation site*	RTCL input (probe) site*	Number of iEEG electrodes	Intervention type (S = sync-stim, M = mixed phase)
1	1	12:15 AM	5	86	b	RPF	mixture of RMH and LMH	10	M
2	2	12:09 AM	7	94	b	LPF	LEC	10	S
3	3	11:30 PM	10	96	b	RPF	RAH	8	S
4	4	2:00 AM	8	118	b	LPF	LPHG	12	S
5	5	12:05 AM	8	55	b	RT	RAH	9	S
6	6	2:09 AM	8	87	u	RT	RAH	10	S
7	7	11:35 PM	10	100	u	RTO	RMH	9	S
8	8	10:50 PM	13	55	u	RPF	RMH	10	M
9	9	11:00 PM	11	90	u	LPF	LAH	10	S
10	10	12:25 AM	10	55	u	LPF	LMH	10	M
11	11	1:50 AM	6	163	u	LPF	RAH	12	M
12	12	11:30 PM	6	45	u	RPF	RAH	13	M
12	13	2:15 AM	8	132	u	RPF	RAH	13	M
13	14	2:35 AM	6	112	u	RPF	RAH	10	S
14	15	3:45 AM	5	59	u	RPF	RMH	9	S
15	16	12:05 PM	4	59	u	RT	RT	11	S
16	17	12:15 AM	5	57	u	LPF	LMH	14	S
17	18	2:00 AM	6	35	u	RPF	RMH	13	S
18	19	12:05 AM	6	55	u	RPF	REC	14	S

Supplementary Table 4. Real-time closed-loop intervention night information

Shaded rows are intervention sessions with full cognitive testing that are also reported in Supplementary Table 2, Fig. 1f,g. Shading color corresponds to stimulation type listed in the rightmost column, sync stimulation in the orbitofrontal cortex (red) or other areas (brown), and mixed-phase stimulation (gray), white background sessions are included in neurophysiology analysis but not cognitive testing. Columns (left to right): (1) Participant ID (Participant #12 participated in two intervention nights); (2) Intervention session number; (3) Number of stimulation blocks per session (4) Total length of session (first stim-block till the end of the last stim-block) (min); (5) Stimulation type – unipolar/bipolar; (6) Stimulation site - hemisphere and approximate location (exact MR scans provided in Extended Data Fig. 2); (7) Location of probe used as input to RTCL system: hemisphere and approximate location in MTL (exact MR scans are provided in Extended Data Fig. 2); (8) Number of iEEG electrodes recorded in session (note that there are 6-8 iEEG contacts per electrode); (9) RTCL mode – sync-stim (red – stimulation site in the prefrontal cortex, brown – in other anatomical areas) /mixed-phase stim (gray). *Abbreviations: R = right hemisphere, L = Left hemisphere; PF = prefrontal cortex, T = temporal cortex, TO = Temporal-occipital cortex, AH=Anterior Hippocampus, MH = Middle Hippocampus, EC = Entorhinal cortex, PHG = Parahippocampal gyrus.
