# Supplementary material for: Augmenting hippocampal–prefrontal neuronal synchrony during sleep enhances memory consolidation in humans
Source: Nat Neurosci. 2023 Jun 1;26(6):1100–10. doi: 10.1038/s41593-023-01324-5 (PMC10244181; doi:10.1038/s41593-023-01324-5)
Supplement: Supplementary file 2 — Reporting Summary [file 41593_2023_1324_MOESM2_ESM.pdf]

nature portfolio

Corresponding author(s): Yuval Nir and Itzhak Fried

Last updated by author(s): Apr 6, 2023

Reporting Summary

Nature Portfolio wishes to improve the reproducibility of the work that we publish. This form provides structure for consistency and transparency in reporting. For further information on Nature Portfolio policies, see our Editorial Policies and the Editorial Policy Checklist.

Statistics

For all statistical analyses, confirm that the following items are present in the figure legend, table legend, main text, or Methods section.

n/a	Confirmed	
☐	☒	The exact sample size (*n*) for each experimental group/condition, given as a discrete number and unit of measurement
☐	☒	A statement on whether measurements were taken from distinct samples or whether the same sample was measured repeatedly
☐	☒	The statistical test(s) used AND whether they are one- or two-sided<br>*Only common tests should be described solely by name; describe more complex techniques in the Methods section.*
☐	☒	A description of all covariates tested
☐	☒	A description of any assumptions or corrections, such as tests of normality and adjustment for multiple comparisons
☐	☒	A full description of the statistical parameters including central tendency (e.g. means) or other basic estimates (e.g. regression coefficient) AND variation (e.g. standard deviation) or associated estimates of uncertainty (e.g. confidence intervals)
☐	☒	For null hypothesis testing, the test statistic (e.g. *F*, *t*, *r*) with confidence intervals, effect sizes, degrees of freedom and *P* value noted<br>*Give P values as exact values whenever suitable.*
☐	☒	For Bayesian analysis, information on the choice of priors and Markov chain Monte Carlo settings
☒	☐	For hierarchical and complex designs, identification of the appropriate level for tests and full reporting of outcomes
☐	☒	Estimates of effect sizes (e.g. Cohen's *d*, Pearson's *r*), indicating how they were calculated

Our web collection on statistics for biologists contains articles on many of the points above.

Software and code

Policy information about availability of computer code

Data collection	We used custom code (Matlab) which is described in detail in the methods section, based on BlackRock Microsystem's freely available API.<br>We will gladly share the code used to track brain states and elicit stimulations upon reasonable request.
Data analysis	Analysis was performed in Matlab 2018b using custom-developed analysis routines.<br>Electrode localization was performed using iELVIS (https://github.com/iELVis/iELVis, employing FreeSurfer v6, BioImage-suite and FSL functions, see links below). When possible, we used published software toolboxes that are reported in the text (wave clus 2, FieldTrip, circstat). We described all custom code in online-methods, provided public GitHub links for custom toolboxes we developed for iEEG analysis and and will share additional code upon reasonable request.<br>FreeSurfer available at https://surfer.nmr.mgh.harvard.edu/fswiki/DownloadAndInstall<br>BioImage-suite software available at https://medicine.yale.edu/bioimaging/suite/lands/<br>iElvis - http://ielvis.pbworks.com/w/page/117734730/Installing%20iELVis<br>FSL (for flirt and slices functions) - http://fsl.fmrib.ox.ac.uk/fsl/fslwiki/<br>https://github.com/mgevasagiv/sleepScoringIEEG - custom Matlab code for sleep scoring based on iEEG data<br>https://github.com/mgevasagiv/rippleDetection_IEEG - custom Matlab code for ripple detection in iEEG data<br>https://github.com/mgevasagiv/epilepticActivity_IEEG - custom Matlab code for IED detection in iEEG data<br>https://github.com/mgevasagiv/sleepOscillations_IEEG - custom Matlab code for slow-wave and spindle detection in iEEG data

For manuscripts utilizing custom algorithms or software that are central to the research but not yet described in published literature, software must be made available to editors and reviewers. We strongly encourage code deposition in a community repository (e.g. GitHub). See the Nature Portfolio guidelines for submitting code & software for further information.

Data

Policy information about availability of data

All manuscripts must include a data availability statement. This statement should provide the following information, where applicable:

- Accession codes, unique identifiers, or web links for publicly available datasets
- A description of any restrictions on data availability
- For clinical datasets or third party data, please ensure that the statement adheres to our policy

Datasets supporting the findings of this paper are available in a Supplementary information file. Source data for figures are provided with this paper.

Human research participants

Policy information about studies involving human research participants and Sex and Gender in Research.

Reporting on sex and gender	We report self-reported gender of 18 participants (11 women, 7 men, 0 other) in the results section and per-participant (Extended Data Table 1). We did not perform gender-based analysis due to the small sample size of our cohort, which is based on unique patient population (see recruitment sub-section below).
Population characteristics	Neurosurgical patients with pharmaco-resistant epilepsy implanted with intracranial depth electrodes for clinical reasons (n=18, ages 19-47y, all fluent English speakers, additional details in Supplementary Table 1).
Recruitment	Neurosurgical patients with pharmacoresistant epilepsy, who met clinical criteria for depth-electrode placement for seizure localization and possible surgical cure by resection of the identified seizure focus, were recruited for cognitive-electrophysiological studies during their hospital stay. Exclusion criteria - non-fluent English. Due to clinical conditions, including length of hospital stay, frequency of seizures, etc., not every patient was able to complete the task.<br>Due to the invasive nature of intracranial depth recordings and stimulation, only participants who were undergoing implantation of electrodes for clinical reasons were recruited. This could present some bias in the data; this possibility is discussed in the main text.
Ethics oversight	UCLA Institutional Review Board

Note that full information on the approval of the study protocol must also be provided in the manuscript.

Field-specific reporting

Please select the one below that is the best fit for your research. If you are not sure, read the appropriate sections before making your selection.

☒ Life sciences ☐ Behavioural & social sciences ☐ Ecological, evolutionary & environmental sciences

For a reference copy of the document with all sections, see nature.com/documents/nr-reporting-summary-flat.pdf

Life sciences study design

All studies must disclose on these points even when the disclosure is negative.

Sample size	Rare data from neurosurgical patients participating in research during sleep were collected over 6 years. We recorded intracranial EEG (iEEG, n = 565 channels) and neuronal spiking activity (n = 325 clusters) from multiple cortical regions in 18 drug-resistant epilepsy patients implanted with depth electrodes for clinical monitoring during 19 stimulation sessions performed during night's sleep. A subset of 16 patients participated in cognitive testing during an additional undisturbed-sleep night.<br>No sample size calculation was performed, but our sample sizes are similar to those reported in previous publications. We are confident that the sample size is sufficient since the main findings are highly significant statistically, and can be observed in data of individual participants. The cognitive performance measure is within-subject to minimize effect of inter-subject variability.
Data exclusions	Two participants with poor memory accuracy scores and one participant with interrupted intervention were excluded from cognitive analysis (criteria detailed in Methods and patient numbers reported in Extended Data Table 1). We excluded channels with high pathological activity from neurophysiological analysis (criteria detailed in Methods).
Replication	The main electrophysiological findings are highly significant statistically and can be observed in data of individual participants. The cognitive performance measure is within-subject to minimize effect of inter-subject variability.
Randomization	Participants were tested during two experimental nights, order counterbalanced and cognitive-test versions randomized between intervention/undisturbed nights (reported in Extended Data table 2), to allow a within-subject control for cognitive testing.<br>Additionally, during a predefined period we assigned patients to a mixed-phase stimulation group, as a control group to the main patient group that underwent synchronizing-stimulation.

Blinding	Patients were blind to the type of stimulation (synchronizing vs mixed phase). Experimenters were blinded to testing condition whenever possible - one experimenter chose the stimulation mode while the others (as well as Neurologist overseeing the experiment) were blind to the mode. Undisturbed night was a night without any interventions so participants and experimenters were aware of the condition.

Reporting for specific materials, systems and methods

We require information from authors about some types of materials, experimental systems and methods used in many studies. Here, indicate whether each material, system or method listed is relevant to your study. If you are not sure if a list item applies to your research, read the appropriate section before selecting a response.

Materials & experimental systems

n/a	Involved in the study
☒	☐ Antibodies
☒	☐ Eukaryotic cell lines
☒	☐ Palaeontology and archaeology
☒	☐ Animals and other organisms
☒	☐ Clinical data
☒	☐ Dual use research of concern

Methods

n/a	Involved in the study
☒	☐ ChIP-seq
☒	☐ Flow cytometry
☐	☒ MRI-based neuroimaging

Magnetic resonance imaging

Experimental design

Design type	*Indicate task or resting state; event-related or block design.*
Design specifications	*Specify the number of blocks, trials or experimental units per session and/or subject, and specify the length of each trial or block (if trials are blocked) and interval between trials.*
Behavioral performance measures	*State number and/or type of variables recorded (e.g. correct button press, response time) and what statistics were used to establish that the subjects were performing the task as expected (e.g. mean, range, and/or standard deviation across subjects).*

Acquisition

Imaging type(s)	*Specify: functional, structural, diffusion, perfusion.*
Field strength	*Specify in Tesla*
Sequence & imaging parameters	*Specify the pulse sequence type (gradient echo, spin echo, etc.), imaging type (EPI, spiral, etc.), field of view, matrix size, slice thickness, orientation and TE/TR/flip angle.*
Area of acquisition	*State whether a whole brain scan was used OR define the area of acquisition, describing how the region was determined.*

Diffusion MRI ☐ Used ☐ Not used

Preprocessing

Preprocessing software	*Provide detail on software version and revision number and on specific parameters (model/functions, brain extraction, segmentation, smoothing kernel size, etc.).*
Normalization	*If data were normalized/standardized, describe the approach(es): specify linear or non-linear and define image types used for transformation OR indicate that data were not normalized and explain rationale for lack of normalization.*
Normalization template	*Describe the template used for normalization/transformation, specifying subject space or group standardized space (e.g. original Talairach, MNI305, ICBM152) OR indicate that the data were not normalized.*
Noise and artifact removal	*Describe your procedure(s) for artifact and structured noise removal, specifying motion parameters, tissue signals and physiological signals (heart rate, respiration).*
Volume censoring	*Define your software and/or method and criteria for volume censoring, and state the extent of such censoring.*

Statistical modeling & inference

Model type and settings	*Specify type (mass univariate, multivariate, RSA, predictive, etc.) and describe essential details of the model at the first and second levels (e.g. fixed, random or mixed effects; drift or auto-correlation).*

Effect(s) tested	*Define precise effect in terms of the task or stimulus conditions instead of psychological concepts and indicate whether ANOVA or factorial designs were used.*

Specify type of analysis: ☐ Whole brain ☐ ROI-based ☐ Both

Statistic type for inference (See Eklund et al. 2016)	*Specify voxel-wise or cluster-wise and report all relevant parameters for cluster-wise methods.*
Correction	*Describe the type of correction and how it is obtained for multiple comparisons (e.g. FWE, FDR, permutation or Monte Carlo).*

Models & analysis

n/a	Involved in the study
☒	☐ Functional and/or effective connectivity
☒	☐ Graph analysis
☒	☐ Multivariate modeling or predictive analysis
